# The Ser/Thr kinase MAP4K4 drives c-Met-induced motility and invasiveness in a cell-based model of SHH medulloblastoma

**DOI:** 10.1186/s40064-015-0784-2

**Published:** 2015-01-14

**Authors:** Karthiga Santhana Kumar, Dimitra Tripolitsioti, Min Ma, Jasmin Grählert, Katja B Egli, Giulio Fiaschetti, Tarek Shalaby, Michael A Grotzer, Martin Baumgartner

**Affiliations:** Department of Oncology, Children’s Research Center, University Children’s Hospital, Zurich, Switzerland; Current address: Department of Biomedicine, University Hospital Basel, Basel, Switzerland; University Children’s Hospital Zürich, Department of Oncology, Children’s Research Center, Neuro-Oncology group, August-Forel Strasse 1, CH-8008 Zürich, Switzerland

**Keywords:** Medulloblastoma, Cancer cell dissemination, Cell motility, c-Met, MAP4K4, Actin dynamics

## Abstract

Medulloblastoma (MB) comprises four molecularly and genetically distinct subgroups of embryonal brain tumors that develop in the cerebellum. MB mostly affects infants and children and is difficult to treat because of frequent dissemination of tumor cells within the leptomeningeal space. A potential promoter of cell dissemination is the c-Met proto-oncogene receptor tyrosine kinase, which is aberrantly expressed in many human tumors including MB. Database analysis showed that c-Met is highly expressed in the sonic hedgehog (SHH) subgroup and in a small subset of Group 3 and Group 4 MB tumors. Using a cell-based three-dimensional cell motility assay combined with live-cell imaging, we investigated whether the c-Met ligand HGF could drive dissemination of MB cells expressing high levels of c-Met, and determined downstream effector mechanisms of this process. We detected variable c-Met expression in different established human MB cell lines, and we found that in lines expressing high c-Met levels, HGF promoted cell dissemination and invasiveness. Specifically, HGF-induced c-Met activation enhanced the capability of the individual cells to migrate in a JNK-dependent manner. Additionally, we identified the Ser/Thr kinase MAP4K4 as a novel driver of c-Met-induced invasive cell dissemination. This increased invasive motility was due to MAP4K4 control of F-actin dynamics in structures required for migration and invasion. Thus, MAP4K4 couples growth factor signaling to actin cytoskeleton regulation in tumor cells, suggesting that MAP4K4 could present a promising novel target to be evaluated for treating growth factor-induced dissemination of MB tumors of different subgroups and of other human cancers.

**Electronic supplementary material:**

The online version of this article (doi:10.1186/s40064-015-0784-2) contains supplementary material, which is available to authorized users.

## Background

Medulloblastoma (MB) is the most common malignant brain tumor in children and accounts for approximately 10% of all pediatric cancer deaths. MB is thought to arise from neuronal progenitor cells harboring defects in the regulation of gene expression that normally controls growth and development of the cerebellum (Roussel and Hatten 2011). MB cells can disseminate from the primary tumor in the cerebellum throughout the central nervous system and cause metastatic disease in as many as 30% of patients at diagnosis. MB comprises a diverse set of tumors (Northcott et al. 2012a) and four molecular subgroups with differential metastatic potential, named WNT (wingless), SHH (sonic hedgehog), Group 3, and Group 4 (Taylor et al. 2012), have been classified, which remain stable from primary to recurrent MB (Ramaswamy et al. 2013). Treatments that specifically target metastatic dissemination are needed to improve patient survival and reduce treatment-related morbidity.

The receptor tyrosine kinase mesenchymal epithelial transition factor (c-Met) is activated by hepatocyte growth factor/scatter factor (HGF), its only known ligand to date, which triggers phosphorylation of Tyr1230, Tyr1234, and Tyr1235 in the intracellular domain of c-Met. c-Met phosphorylation promotes the induction of various intracellular signaling pathways (Trusolino et al. 2010) to control cell proliferation, survival, and mobilization through the regulation of integrin function and cytoskeleton dynamics (Trusolino et al. 2010). Aberrant c-Met activation occurs in various human cancers in different organs, including the brain, and is associated with disease progression and metastatic dissemination (Sierra and Tsao 2011; Li et al. 2005; Joo et al. 2012).

c-Met is expressed in surgical MB specimens and MB cell lines and its expression and the expression of its ligand HGF is associated with significantly worse outcome in patients (Li et al. 2005). Along with SHH, increased expression of HGF promotes formation and growth of MB tumors in mice (Binning et al. 2008). An increased level of HGF was found sufficient to drive invasiveness of orthotopically xenografted DAOY MB cells (Li et al. 2005). No activating mutation has been reported for MB-expressed c-Met to date, whereas increased c-Met activity has been linked to proliferation, anti-apoptosis, and migration in MB (Li et al. 2005; Provencal et al. 2009; Guessous et al. 2012; Guessous et al. 2010; Kongkham et al. 2010; Onvani et al. 2012). c-Met was found to increase the expression of the transcription factor v-myc avian myelocytomatosis viral oncogene homolog (MYC) (Li et al. 2008), which is the hallmark of the most aggressive form of MB (Taylor et al. 2012). Pro-metastatic functions of c-Met are supported by the hyaluronan (HA) receptor CD44 and in particular by its transcript variant CD44v6, which supports c-Met-dependent signaling (Orian-Rousseau et al. 2002). Although CD44 expression has been associated with WNT and SHH signaling in MB, it’s expression has not yet been analyzed in MB (Katoh and Katoh 2009; Asuthkar et al. 2012).

The molecular mechanisms and downstream effectors that mediate HGF-induced MB cell dissemination are incompletely understood. Herein we used cell-based *in vitro* two- and three-dimensional (2D/3D) motility assays combined with live-cell imaging and biochemical approaches to investigate and characterize potentially druggable mediators of HGF-c-Met-induced MB cell dissemination.

## Results

### c-Met and its co-receptor CD44 are highly expressed in a subset of MB tumors and patient derived cell lines

**Figure 1:**
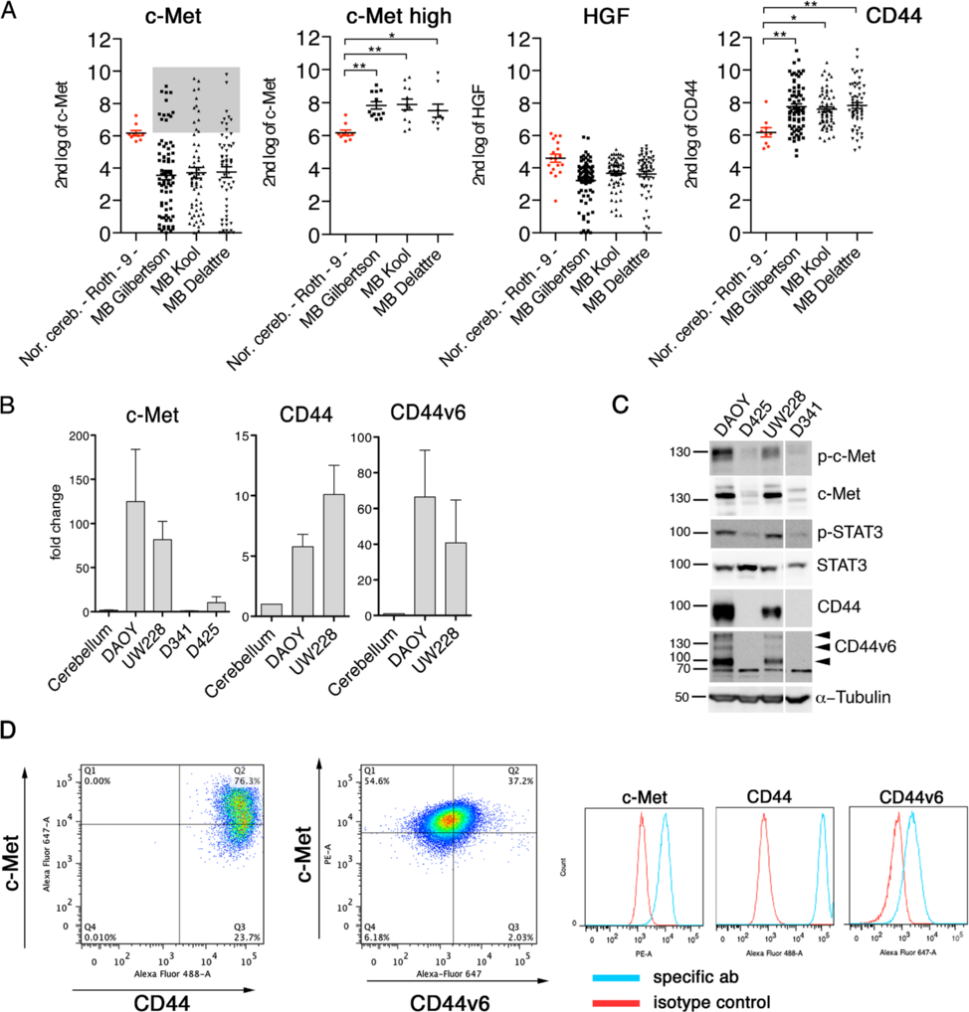
**Expression of c-Met in medulloblastoma clinical samples and cell lines. (A)** Expression analysis of c-Met, HGF and CD44 in three different MB tumor collections (n_total_ = 195) and in normal adult cerebellum (n = 9). **(B)** Comparative quantitative real-time PCR expression analysis of c-Met, CD44 and CD44v6 in established MB cell lines and adult cerebellum sample. **(C)** Expression and activation analysis of the c-Met pathway, CD44, and CD44v6 by immunoblotting (IB) in four different MB cell lines using the antibodies indicated to the right of the blots. **(D)** Flow cytometry analysis of DAOY cells quantifying surface expression of c-Met, CD44, and CD44v6. Dot plots compare expressions of c-Met and CD44 or c-Met and CD44v6. Histograms show relative fluorescence intensities of isotype control and specific antibody samples.

To determine the potential clinical relevance of c-Met in larger cohorts of MB, we compared the mRNA expression levels of c-Met in the Gilbertson, the Kool and the Delattre datasets available through the R2 platform for visualization and analysis of the microarray data. As control, we used nine cerebellum samples of patients aged between 23 and 50 years. We found that the median mRNA level of c-Met and its ligand HGF in MB tumors from these three different primary sample cohorts were clearly below that of normal human cerebellum (Figure 1A). However, a sub-population of MB tumors averaging 17.5% (Figure 1A, c-Met high) showed significantly increased c-Met expression. Moreover, the same datasets revealed high mRNA expression of the c-Met co-receptor CD44 (Orian-Rousseau et al. 2002) in all MB tumor samples. By analyzing 103 primary MB tumors of the Northcott 103 dataset (Northcott et al. 2011), Onvani *et al.* described the association of c-Met with the SHH subgroup (Onvani et al. 2012). We confirmed this finding using the 285 tumors of the MAGIC dataset (Northcott et al. 2012b) (Additional file 1: Figure S1A). An analogous but less marked association was also observed for HGF (Additional file 1: Figure S1B), but not for CD44 (Additional file 1: Figure S1C). Using quantitative real-time PCR (Figure 1B) and immunoblotting (IB) approaches (Figure 1C), we detected high c-Met, CD44, and CD44v6 expression both at the mRNA and protein levels in DAOY and UW228 cell lines, and much less (c-Met) or no (CD44/CD44v6) expression in D341 and D425 cell lines. Interestingly, three bands were detected in the anti-CD44v6 blot (Figure 1C, arrowheads), suggesting the presence of different CD44 isoforms with incorporated v6 variable region. DAOY cells are sensitive to sonic hedgehog (Gotschel et al. 2013) and considered a SHH-like MB cell line, whereas D341 is considered a group 3 cell line (Snuderl et al. 2013). We confirmed surface expression of c-Met, CD44, and CD44v6 on DAOY (Figure 1D) and UW228 cell lines (not shown) by flow cytometry. This analysis revealed that >90% of DAOY cells expressed c-Met, 100% expressed CD44, while only approximately 40% expressed the CD44v6 isoform. We therefore continued our studies by focusing specifically on c-Met and by studying what effects c-Met activation by its ligand HGF may have on cell migration and invasion and which effector pathways are needed to mediate the c-Met responses.

### HGF stimulation activates JNK and MAPK/ERK pathways and promotes motility

**Figure 2:**
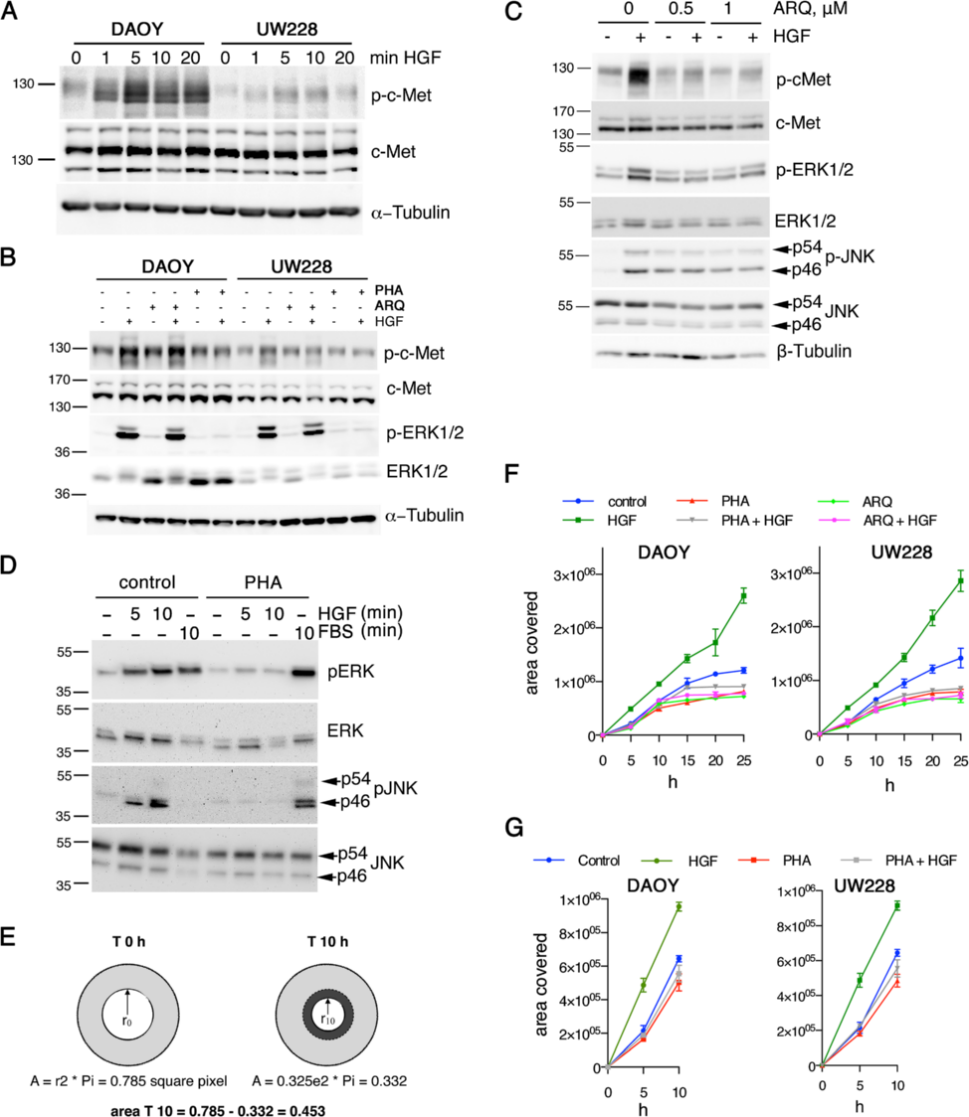
**c-Met is activated by HGF in medulloblastoma cells and promotes wound closure. (A)** IB of DAOY and UW228 lysates of cells after HGF stimulation. Antibodies used as indicated to the right of the panels. **B)** IB analysis of PHA-665752 and ARQ 197 (1 μM, 2 h pretreatment) effects on HGF-induced (50 ng/mL, 10 min) c-Met and ERK pathway activation (anti-p-c-Met and anti-pERK, respectively). **(C)** IB analysis as in B of the effect of prolonged (24 h) pretreatment with ARQ197 inhibitor on HGF-induced c-Met and ERK activation in UW228 cells. **(D)** IB analysis of HGF-induced activation of JNK and ERK in DAOY cells (20 ng/mL HGF −/+ 250 nM PHA-665752). **(E)** Schematic showing how the area covered by the cells in the Oris migration assay was determined. Light grey: cell monolayer at T_0h_. White: gap after removal of the plug. Dark grey: area covered by cells at T_10h_. **(F)** Quantification (means + S.D.) of Oris migration assays using DAOY or UW228 cells in HGF-treated (20 ng/mL) medium containing 0% FCS and pharmacological inhibitors (PHA-665752 and ARQ 197, both at 125 nM). **(G)** As F) but progression of gap closure shown for 0–10 h only.

To determine dynamics of c-Met-induced ERK and JNK activation in DAOY and UW228 cells, we stimulated the cells with HGF in a time course experiment. We found that HGF stimulation of DAOY or UW228 cells promotes rapid phosphorylation of c-Met (IB p-c-Met) within five to ten min (Figure 2A) and the concomitant activation of the downstream effector extracellular-signal-regulated kinase (ERK, IB p-ERK) (Figure 2B). c-Met and ERK phosphorylations were blocked when the cells were pretreated for 2 h with the ATP-competitive c-Met inhibitor PHA-665752 (Christensen et al. 2003) but not with the non ATP-competitive inhibitor ARQ197 (Munshi et al. 2010) (Figure 2B). However, we found that 24 h ARQ197 pretreatment was necessary to block acute, HGF-induced c-Met signaling (Figure 2C). c-Met can activate the c-Jun N-terminal kinase (JNK) (Rodrigues et al. 1997), which controls growth and invasion of MB cells (Zavarella et al. 2009). Consistently, we detected PHA-665752-sensitive phosphorylation of mainly the p46 isoform of JNK within five to ten minutes of HGF stimulation (Figure 2D). Interestingly, 24 h treatment with ARQ197 (Figure 2C) also caused increased JNK phosphorylation by an unknown mechanism, which was not further increase by HGF stimulation, because c-Met activity was blocked. To determine whether HGF stimulation and/or c-Met inhibition affected cell viability and/or proliferation, we performed a tetrazolium salt WST assay on DAOY and UW228 cells treated with various combinations of HGF and PHA-665752 or ARQ197. Corresponding to c-Met expression levels (high in DAOY and UW228, low in D425), proliferation/viability was effectively reduced by the c-Met inhibitors in DAOY and UW228 cells and only moderately affected in D425 cells (Additional file 2: Figure S2). To monitor HGF-induced cell migration, we used the Oris migration assay (Gough et al. 2011) (Figure 2E) and measured the effect of HGF-c-Met signaling on the cells’ capability to close a circular gap created by the insertion of a rubber stopper into the well that prevented cell attachment and growth (Figure 2F). Using time lapsed video microcopy imaging, we found that HGF treatment significantly accelerated gap closure within 24 h - both under serum-free (Figure 2F) and 10% serum (Additional file 3: Figure S3A) conditions. Importantly, time-lapse imaging showed that HGF treatment strikingly increased migration already within 5 h of incubation (Figure 2G). In DAOY cells, PHA-665752 treatment in the absence of ectopically added HGF reduced gap closure by nearly 50%, suggesting that an endogenous or a serum-derived factor activates the c-Met signaling axis and promotes pro-migratory signals (Additional file 3: Figure S3B). Overall, we showed that c-Met signaling was active in MB cells, that it was further activated by the exogenous addition of HGF and that it contributed to cell migration on 2D surfaces.

### HGF promotes single cell motility and invasiveness

**Figure 3:**
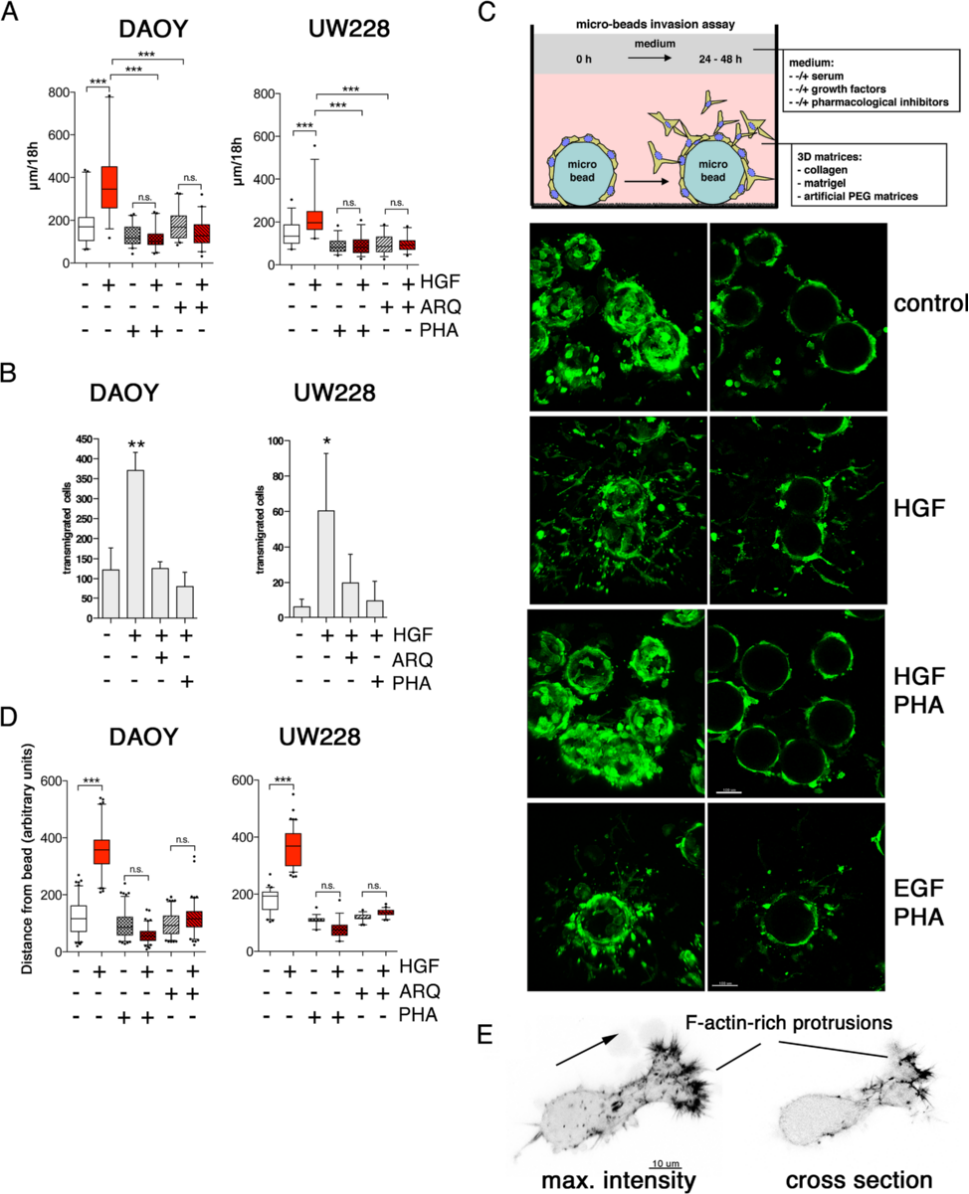
**HGF promotes invasive motility of single medulloblastoma cells. (A)** Single cell motility of DAOY and UW228 cells was measured using live cell imaging (HGF: 20 ng/mL, ARQ197 and PHA-665752 250 nM). Box plots of three independent experiments are shown. **(B)** Boyden chamber invasion assay under conditions as described in (A). Mean total numbers of cells transmigrated and S.D. of representative triplicate experiment are shown. Statistical analysis: T-test, * = 0.0454, ** = 0.0038. **(C)** Upper: schematic of microbead invasion assay setup. Lower: microbeads coated with DAOY cells were embedded in collagen and cells were allowed to disseminate for 24 h. Confocal microscopy analysis of LA-EGFP fluorescence 24 h after embedding is shown (left: maximum intensity projection of Z-stacks, right: single cross-section through middle of beads). **D)** Quantification of mean and range of cell dissemination from microbeads shown in C (triplicate measurements, ten beads quantified per measurement, dot plot with SD). **E)** High-resolution confocal images of an HGF-induced (20 ng/mL) LA-EGFP expressing DAOY cell migrating in collagen. F-actin distribution is shown as inverted grey scale. Arrow: direction of migration. Note high F-actin content in invasive protrusions at leading edge of the cell.

In assays that measure the area covered by cells such as wound healing assays or end-point Oris migration assay, it is not possible to discriminate between individual cell migration and proliferation. To determine whether HGF-induced c-Met activation indeed caused increased cell motility, we determined the speed of single cells. Towards that end, we measured the pathlength of single cells that migrated over a given time (speed) by time-lapse video microscopy. We found that HGF promoted a twofold increase in cell speed both in DAOY and UW228 cells (Figure 3A), which was blunted when c-Met was pharmacologically inhibited by either PHA-665752 or ARQ197. HGF also significantly increased single cell motility in the matrigel invasion in a c-Met-dependent manner (Figure 3B). However, the matrigel invasion assays does not permit monitoring the behavior of single cells inside a 3D matrix and measuring their speed of migration. To solve that problem, we developed a versatile micro bead invasion assay for MB cells and assessed cell dissemination from the beads into the surrounding collagen. Importantly, cells migrating inside the matrix are fully accessible for fixed- and live-cell microscopy (Figure 3C, upper). We found that HGF or epidermal growth factor (EGF) treatment promoted massive cell dissemination (Figure 3C and D). As expected, PHA-665752 prevented HGF- but not EGF-induced dissemination, confirming the specificity of this compound for the c-Met receptor tyrosine kinase. We observed that cells migrating in the collagen matrix displayed marked, F-actin rich invasive protrusions at the leading edges (Figure 3E), suggesting that local F-actin polymerization in the lamellipodia of cells is instrumental for motility. Taken together, these data demonstrate that HGF triggers dissemination of MB cells in 2D and 3D environments by accelerating motility at the single cell level. We furthermore detected enhanced local F-actin polymerization, suggesting F-actin turnover acting at the leading edge in HGF-stimulated cells as driving force.

### JNK and MAP4K4 are downstream effectors of HGF-induced motility

**Figure 4:**
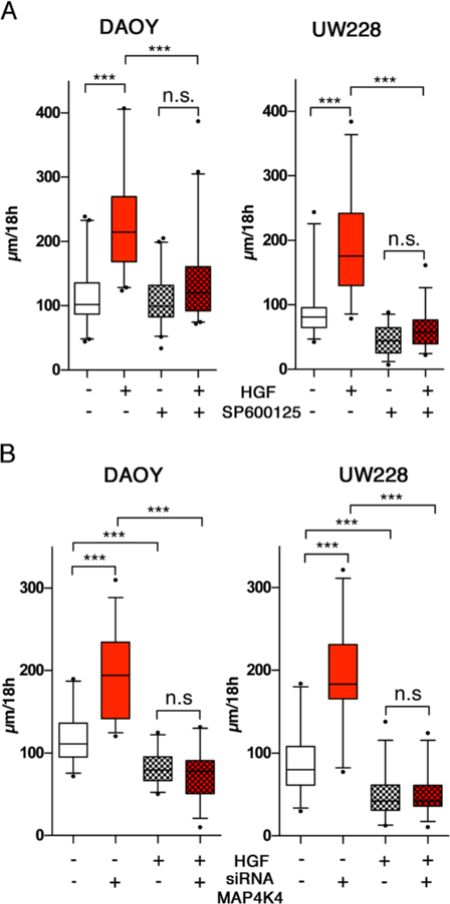
**HGF-induced single cell motility is mediated by the Ser/Thr kinases JNK and MAP4K4. (A)** Motility of single cells migrating on flat 2D surfaces in the absence or presence of HGF (20 ng/mL) and the JNK inhibitor SP665757 (10 ng/mL). Box plots of three independent experiments are shown. **(B)** Same experimental approach as in (A) but instead of using a pharmacological inhibitor, MAP4K4 was depleted from cells using validated MAP4K4-specific (+) siRNA. (−) is a negative control siRNA .

JNK is highly expressed in the brain and controls neuronal cell migration during development (Zdrojewska and Coffey 2014) and in MB cells, HGF stimulation promoted JNK activation (Figure 2D). To test whether JNK activity was necessary for HGF-induced motility, we treated MB cells with the JNK inhibitor SP600125 (Han et al. 2001). We found that HGF-stimulated single cell motility (speed) was markedly reduced when JNK activity was blocked (Figure 4A). Interestingly, the ablation of JNK activity in the absence of HGF significantly reduced speed of single UW228 but not DAOY cells, indicating that serum-dependent motility bypasses JNK in DAOY but not in UW228 cells (Figure 4A) and suggesting different JNK pathway regulation in these closely related cell lines. We confirmed the sensitivity of HGF-induced single cell motility to JNK inhibition with the two additional JNK inhibitors JIP-1 (153–163) and AEG 3482 (Additional file 4: Figure S4). One upstream kinase of the JNK signaling pathway is the Ser/Thr kinase mitogen-activated protein kinase kinase kinase kinase 4 (MAP4K4) (Su et al. 1997). MAP4K4 mediates HGF effects on anchorage-independent growth and invasiveness (Wright et al. 2003), promotes F-actin dynamics in lamellipodia and cell motility (Baumgartner et al. 2006; Ma and Baumgartner 2014) and contributes to the progression of solid tumors in humans (Collins et al. 2006; Hao et al. 2010; Liang et al. 2008; Liu et al. 2011; Qiu et al. 2012). In human MB samples of all four subgroups, MAP4K4 is highly expressed, most significantly in the SHH and Group 4 subgroups (Additional file 1: Figure S1D). Depletion of MAP4K4 using a small interfering RNA (siRNA) approach abrogated the pro-migratory effect of HGF and also significantly reduced steady-state motility (Figure 4B). Thus, HGF-Met signaling increases speed of single migrating cells through mechanisms requiring JNK activity and MAP4K4 function, suggesting that these two kinases are essential regulators of MB cell dissemination.

### HGF promotes cortical actin polymerization and membrane protrusion

**Figure 5:**
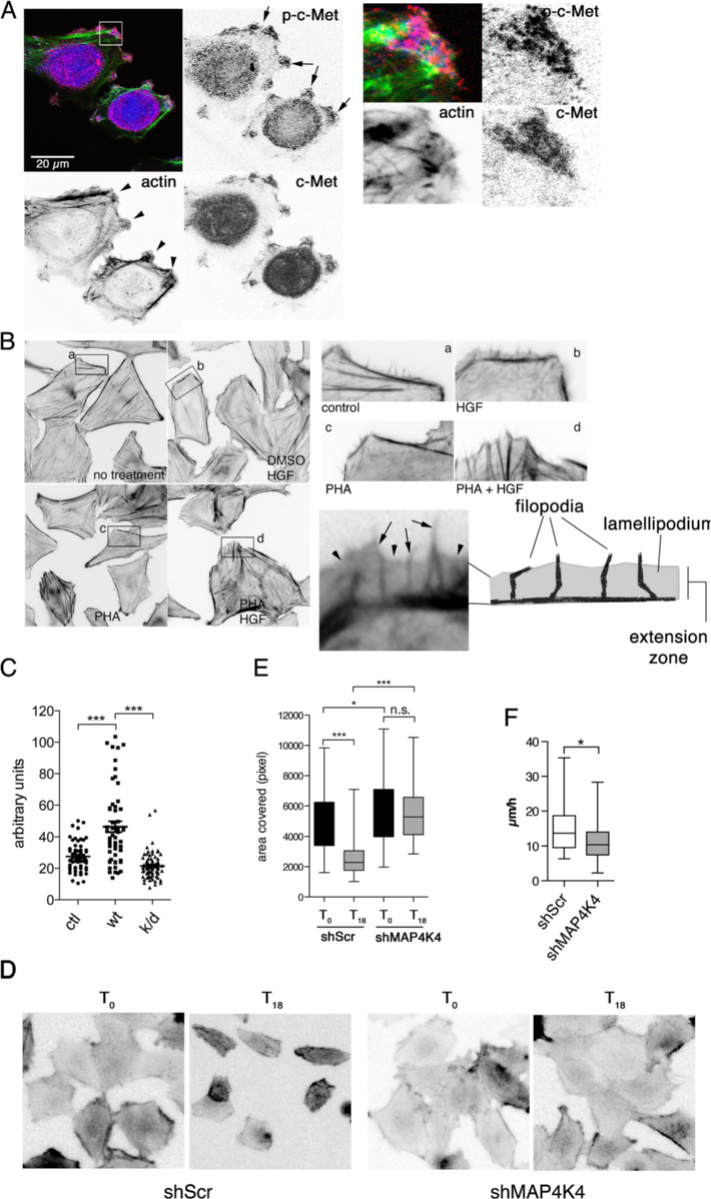
**HGF promotes cortical actin dynamics in medulloblastoma cells. (A)** Immunofluorescence analysis (IFA) of c-Met and phosphorylated c-Met (p-c-Met) localization in lamellipodia of DAOY cells. Color overlay and inverted grey-scale images of p-c-Met (red), actin (green) and c-Met (blue) are shown. Magnifications are 4× of boxed area in overlay. Arrows indicate c-Met-rich lamellipodia. **(B)** IFA of Alexa-488-phalloidin-stained F-actin cytoskeletons in un-stimulated and HGF-stimulated (20 ng/mL, t = 10 min) DAOY cells, −/+ PHA-665752 (500 nM). Inverted grey-scale images of Alexa-488-phalloidin fluorescence are shown. Magnifications are 4× of boxed areas. Lower left magnification is 4× of sheet-like protrusion in b). Arrows: filopodia, arrowheads: leading edge of F-actin sheet (extension zone, see schematic). **(C)** F-actin dynamics in DAOY cells transfected with LA-mCherry and either enhanced green fluorescent protein-tagged, wild-type (wt) or kinase-defective (k/d) MAP4K4 were recorded by confocal live cell microscopy imaging. See Additional file 5: Figure S5 for still images of representative cells. Dot blots show protrusion lengths in control cells or cells expressing either EGFP-MAP4K4-wt or EGFP-MAP4K4-k/d. **(D)** Still images of time-lapse movies of DAOY-LA-EGFP-shScr or DAOY-LA-EGFP-shMAP4K4_1 cells stimulated with HGF (20 ng/mL). T_0_ is 0 h and T_18_ is 18 h after HGF stimulation. Inverted grey-scale of LA-EGFP fluorescence (F-actin cytoskeleton) is shown. **(E)** Cells were treated as described in (D). Box plots of areas in pixels covered by individual cells quantified at T_0_ and T_18_. **(F)** Box plot of speeds of single sh control or shMAP4K4 cells in the presence of HGF. Statistical analysis: T-test (*: P = 0.0208).

Increased F-actin dynamics and cell motility indicated that c-Met could be active in lamellipodia to control F-actin dynamics in these structures. We used immunofluorescence (IF) microscopy to localize c-Met and p-c-Met in MB cells. Indeed, in lamellipodia of DAOY (Figure 5A, arrowheads) and UW228 (Additional file 5: Figure S5A) cells, we detected accumulations of c-Met and p-c-Met (Figure 5A, arrows). To test whether c-Met activation promoted cortical actin dynamics (Rottner and Stradal 2011), we stimulated MB cells with HGF and monitored immediate and late changes in cortical F-actin by immunofluorescence analysis (Figure 5B) and live cell imaging (movies Additional file 6: SM1, Additional file 7: SM2, Additional file 8:SM3), respectively. Interestingly, within 15 min we observed *de novo* synthesis of lamellipodial branched F-actin in the extension zone in HGF-stimulated cells (Figure 5B, magnifications), which was prevented when cells were pretreated with PHA-665752. We also observed accelerated and more prominent cortical F-actin turnover in HGF-stimulated UW228 cells (movies Additional file 6: SM1, Additional file 7: SM2, Additional file 8: SM3). To test whether MAP4K4 could promote cortical F-actin dynamics in MB cells, we expressed either enhanced green fluorescent protein (EGFP)-tagged wild-type (EGFP-MAP4K4-wt) or a kinase-defective (EGFP-MAP4K4-k/d) mutant of MAP4K4 in DAOY cells together with Lifeact fused to mCherry (LA-mCherry). We monitored F-actin dynamics by confocal live cell microscopy and quantified morphodynamic alterations of cell protrusions by kymography (Figure 5C and Additional file 5: Figure S5B). We found that F-actin polymerization dynamics in lamellipodia were significantly higher in cells expressing MAP4K4-wt and blunted in cells expressing MAP4K4-k/d. Interestingly, cells depleted of MAP4K4 by inducible short hairpin RNA expression (shRNA, see below) were also no longer able to respond to HGF stimulation with scattering (Figure 5D) and morphological alterations (contraction, measured as area covered per cell, 5E). Specifically, we observed that HGF-induced cell scattering evident in a culture of semi-confluent cells 24 h after HGF stimulation and resulting in dissociated cells with few cell-cell contacts, was abrogated by MAP4K4 depletion using shRNA. Reduced scattering may in part be due to reduced motility of single cells (Figure 5F). However, in shRNA MAP4K4-expressing cells, we also observed more cells with intact cell-cell contacts, suggesting that MAP4K4 effects on cell dissemination impact different levels of cell migration control. Taken together, our data show that MAP4K4 orchestrates HGF-induced morphodynamic processes and MB cell motility by controlling F-actin cytoskeleton dynamics and its depletion reduces the capability of MB cells to scatter in response to HGF.

### MAP4K4 promotes HGF-induced single cell scattering and collagen invasion

**Figure 6:**
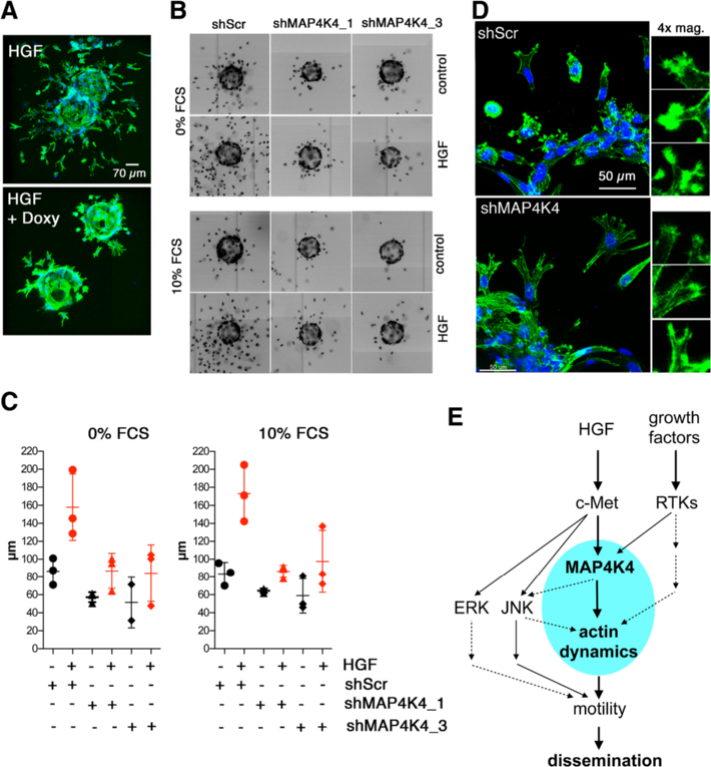
**MAP4K4 drives HGF-induced cell dissemination in fibrillar collagen. (A)** Z-stack maximum intensity projection of collagen embedded, disseminating DAOY-doxy-MAP4K4 cells. Short hairpin RNA expression was induced by doxy stimulation for 48 h before the start of the experiment (lower panel). Cells were stimulated with HGF (20 ng/mL) for 24 h. Green: F-actin, blue: DNA. **(B)** Representative montaged images of inverted grey-scale fluorescence of nuclear DNA to visualize cell dissemination. shRNA control or shMAP4K4_1/_3 expression with doxycycline and FCS and HGF treatments as indicated. **C)** Quantification of average velocities of disseminating cells with treatments as indicated. The averages of three independent experiments and S.D. are shown. **D)** High resolution confocal Z-stack of invading shScr and shMAP4K4 LA-EGFP cells. Note reduced F-actin in invasive protrusions of shMAP4K4 cells **E)** Schematic representation of signaling pathways investigated. Highlight blue is the central MAP4K4 controlled machinery that we propose to drive the dynamic remodeling of the actin cytoskeleton required for cell dissemination downstream of growth factor signaling.

To test whether MAP4K4 was driving HGF-induced invasive motility in collagen, we used MB cells expressing doxycycline (doxy)-inducible scrambled control shRNAs (shScr) or shRNAs targeting MAP4K4 (shMAP4K4) (Additional file 5: Figure S5C) in the micro bead invasion assay. Confocal microscopy imaging showed that HGF-promoted dissemination was markedly reduced in MAP4K4-depleted cells (Figure 6A). To quantify invasiveness of larger numbers of cells, we visualized cell nuclei (Figure 6B) and measured the distance between the bead and the individual nuclei (Figure 6C). We found that HGF-induced single cell dissemination in 3D was significantly reduced when MAP4K4 was depleted, both in 0% and 10% FCS medium. Importantly, HGF-stimulated shScr cells displayed considerably higher F-actin content at the leading edge than did MAP4K4-depleted cells (Figure 6D), indicating that MAP4K4-induced F-actin polymerization activity (Figure 5C–E) was also needed for forming invasive protrusions during cell migration in collagen. In conclusion, HGF promoted MB cell dissemination in collagen is driven by MAP4K4, probably by triggering the invasive, F-actin-rich membrane protrusions required for cells to invade and migrate (Figure 6E).

## Discussion

In this study, we have investigated the functional significance of the HGF-c-Met signaling pathway for MB cell dissemination. We found that c-Met expression is upregulated in the SHH subgroup and in a subset of Group 3 and Group 4 MB tumors, as well as in some established SHH MB laboratory cell lines. We demonstrated that c-Met activation by its ligand HGF promotes single cell motility of MB cells and their invasion into Matrigel and 3D collagen gels. We further showed that HGF-induced motile and invasive cell behavior requires the Ser/Thr kinase MAP4K4, which controls F-actin cytoskeleton dynamics in cellular protrusions necessary for motility and invasiveness. Thus, our studies reveal a novel, growth factor-dependent signaling circuit that promotes MB cell dissemination through MAP4K4-dependent cytoskeleton regulation, and underscore the necessity of patient stratification based on growth factor sensitivity of the tumor for rational targeting of cancer promoting signaling pathways.

Others and we have revealed a striking association of c-Met expression with SHH MB ((Onvani et al. 2012) and Additional file 1: Figure S1A) and we found that c-Met is overexpressed in approximately 18% of MB tumors compared to cerebellum controls. It is possible that c-Met could contribute to tumor progression by causing dissemination of the subset of recurrent SHH tumors reported recently (Ramaswamy et al. 2013). Importantly, c-Met function could also contribute to MB tumor cell dissemination in other subgroups by driving cell motility. However, other cellular parameters such as the capability to survive in the CSF or to colonize the new niche will be as important as well, and which could explain the discrepancy in the relative clinical outcomes between c-Met-high SHH and for example c-Met-low Group 3 tumors. Future studies examining large cohorts of patients in a subgroup-specific manner will now be required to fully appreciate the role of c-Met signaling in this context. Although the expression of the c-Met co-receptor CD44 was high in all MB tumor samples analyzed, its role in MB is unclear and further studies will also be needed here to reveal c-Met-related and un-related effects of CD44 in MB pathogenesis. Unlike CD44 expression in tumor samples, CD44 expression in MB cell lines was restricted to those expressing c-Met. Of these, only 40% co-expressed also the HGF-c-Met-interacting variant isoform CD44v6. In glioblastoma, CD44 expression conferred growth advantages and therapeutic resistance (Xu et al. 2010) and it remains to be resolved whether analogous mechanisms are also active in MB, in particular in the context of c-Met interaction with CD44v6.

Several earlier studies have implicated a role of HGF-c-Met in MB growth and dissemination and scratch wound healing assays revealed the involvement of c-Met in wound closure (Kongkham et al. 2010). However, it remained unclear whether c-Met inhibition reduced MB cell dissemination because it impaired proliferation or because it impaired cell motility. We clarified this point by providing direct evidence that HGF-c-Met function promotes the capability of MB cells to migrate, which ultimately accelerates their dissemination both in 2D and 3D environments. It can be assumed that the dual function of c-Met, stimulation of proliferation and of single cell motility is effective in other cell types or tumor cells expressing high c-Met and explains in part the effective tumorigenic activity of this receptor.

How c-Met-induced JNK promotes MB cell motility is not known; it is possible that JNK is relevant in MB cells for proper function of the microtubule skeleton during motile processes through its activity toward the microtubule regulatory proteins superior cervical ganglion 10 (SCG10), doublecortin (DCX) (Zdrojewska and Coffey 2014) or microtubule-associated protein 1b (MAP1b) (Yamasaki et al. 2011). In addition to JNK, we identified MAP4K4 as a novel kinase essential for efficient dissemination of MB cells. MAP4K4 and its murine (Nck-interacting kinase), fly (misshapen) and worm (MIG15) orthologs are evolutionary conserved and control migration of both neurons (Chapman et al. 2008; Poinat et al. 2002; Shakir et al. 2006; Teuliere et al. 2011) and cancer cells (Collins et al. 2006; Wright et al. 2003; Hao et al. 2010; Liang et al. 2008; Liu et al. 2011; Qiu et al. 2012). Although its function downstream of HGF has been suggested (Wright et al. 2003), our findings are the first to demonstrate the involvement of MAP4K4 downstream of c-Met in tumor cells. Depletion of MAP4K4 reduced dissemination and the accumulation of F-actin in focal invasion structures. This finding is consistent with established functions of MAP4K4 as a regulator of cortical actin dynamics (Baumgartner et al. 2006; Poinat et al. 2002; Teuliere et al. 2011; Ma and Baumgartner 2014; Wright et al. 2003; Yan et al. 2001) and it is thus conceivable that MAP4K4 triggers and coordinates spatio-temporal actin polymerization and turnover, both of which are essential for efficient cell movement. Thus, MAP4K4 function is likely needed at the single cell level to trigger invasive cell protrusions, which in turn are necessary for motility and invasiveness of MB tumors. Although MAP4K4 is an established upstream activator of JNK (Machida et al. 2004), we could not provide convincing evidence that MAP4K4 is active in this function in MB cells as well (not shown). Hence, we concluded that while both kinases are essential for MB motility, they do probably act in parallel pathways rather than in a serial one. Considering that MAP4K4 is activated by various growth factors including HGF, PDGF (Yan et al. 2001), TNFα (Yao et al. 1999) or integrin activation (Poinat et al. 2002), we assume that several different receptor-mediated pathways trigger MAP4K4-dependent MB cell dissemination. Consequently, MAP4K4 could act as a hub to divert extracellular derived cues toward morphodynamic processes promoting motility and invasiveness (Figure 6E). Thus, we now need to further refine our understanding of upstream activators and downstream effectors of MAP4K4 in MB, because of its potential significance as a druggable anti-metastatic target for a recently synthetized novel MAP4K4 inhibitor (Crawford et al. 2014).

In summary, we have shown that the HGF-c-Met signaling pathway promotes MB cell dissemination by enabling cell dissociation, rapid movement and efficient matrix invasion of single cells. We revealed the implication of the Ser/Thr kinase MAP4K4 and its cytoskeleton modulatory functions and suggest it as a potential novel anti-metastatic target worth to investigate further. Finally, the pro-migratory functions of MAP4K4 through cytoskeleton regulation revealed herein might contribute to the metastatic progression of SHH subgroup and other MB tumors where MAP4K4 is overexpressed.

## Conclusions

We have established a novel, cell-based assay to monitor cancer cell dissemination in three-dimensional matrices. We show for the first time that HGF-induced c-Met activation enhanced the speed of migration of the individual Medulloblastoma cells and show that the Ser/Thr kinase MAP4K4 is an essential mediator in this process. We conclude that MAP4K4 couples growth factor signaling to actin cytoskeleton regulation in tumor cells, suggesting that MAP4K4 could be a promising novel target to be evaluated for treating growth factor-induced dissemination of Medulloblastoma tumors of different subgroups and of other human cancers.

## Methods

### Ethics statement

This work was conducted according to the ethical guidelines of the University of Zürich. No donor material was used.

### Expression analysis using R2 database

All data used are accessible through the open access platform R2 for visualization and analysis of the microarray data (http://r2.amc.nl). The following datasets were used: Delattre 54 MAS 5.0 – u133p2 (54 MB samples), Gilbertson 76 MAS 5.0 – u133p2 (76 pediatric MB samples, PubMed link: 22722829), Kool 62 MAS 5.0 – u133p2 (62 human MB samples, PubMed link 18769486), Northcott 103 rma_sketch – huex10t (103 primary MB samples, PubMed link 20823417) and MAGIC 285 rma-sketch – hugene11t (285 primary MB samples, PubMed link 22832581) Analysis was performed as described in (Fiaschetti et al. 2014). The nine normal cerebellum samples are from human subject aged as follows: Donor 1–25 year old male; donor 2–38 year old male; donor 3–39 year old female; donor 4–30 year old male; donor 5–35 year old male; donor 6–52 year old male; donor 7–50 year old female; donor 8–48 year old female; donor 9–53 year old female; donor 10–23 year old female.

### Reagents

HGF: 0.25 μM = 20 ng/mL (Preprotech), JIP-1 (153–163) (1565, Tocris), ARQ 197 (A-1109, Active Biochemicals), PHA-665752 10 μM, AEG 3482 5 μM (Axon), (Selleck Chemicals, 10 μM). SP600125 20 μM (S5567), Doxycycline (44577) Blasticidin (15205) (Sigma-Aldrich), AEG 3482 (1291, Axon Medchem).

### Cell culture

DAOY, UW228-2, D341, and D425 cells were grown as described in (Fiaschetti et al. 2014). DAOY-LA-EGFP were generated by lentiviral transduction of DAOY with cells pLenti-LA-EGFP.

### Transfection

5 × 10^5^ cells/well were seeded in 6-well plates and 24 h later transiently transfected using Jet-Pei (101–10 Polyplus), with 2.5 μg of plasmids expressing LA-mCherry (pLenti-LA-mCherry) and either MAP4K4-wt (pEGFP-C2 NIKwt) or MAP4K4-kinase dead (pEGFP-C2 NIKD152N) (Baumgartner et al. 2006).

### Immunoblotting

RIPA buffer lysates were probed with the following primary and secondary antibodies: phospho-c-Met (44888, Life Technologies), c-Met (3148), phospho-STAT3 (9131), STAT3 (9132), phospho-JNK (4668), JNK (9258), phospho-ERK1/2 (9101), ERK1/2 (9102), CD44 (3578) (Cell Signaling), anti-MAP4K4 (80418, Abcam), α-tubulin (T9026, Sigma-Aldrich), and CD44v6 (MAB4073, clone VFF-18, Millipore), anti-mouse horseradish peroxidase (HRP)-linked (7076) and anti-rabbit HRP-linked (7074) (Cell Signaling). Primary antibodies were diluted 1:1000 except for α-tubulin (1:40000). Secondary antibodies were diluted 1:2000.

### Immunofluorescence analysis

Cells were fixed and treated as described in (Ma and Baumgartner 2014). Primary antibodies were diluted 1:200 and incubated overnight at 4°C: α-phospho-c-Met (#44888, Life Technologies), c-Met (3148), CD44 (3578) (Cell Signaling), α-tubulin (T9026, Sigma-Aldrich), Alexa488- (A12379, Life Technologies), Cy3- (711-165-152), and Cy5-coupled (415-175-166) secondary antibodies were used (Jackson Immuno Research). Secondary antibodies and tetramethylrhodamine isothiocyanate-coupled phalloidin (Sigma-Aldrich) were diluted 1:500. Images were acquired on an Axioskop 2 mot plus fluorescence microscope (Zeiss).

### Confocal live cell imaging

DAOY and UW228 cells stably expressing LA-EGFP were seeded in serum-free HEPES-buffered (25 mM) medium overnight on ibidi 8-well slides (5000 cells/well). PHA-665752 (500 nM) was added 1 h prior to and HGF (20 ng/mL) was added at the start of image acquisition in SP8 Leica confocal microscope. A 63× water immersion objective was used to acquire 60 Z-stacks of six images of EGFP fluorescence/timepoint (15 s intervals, 15 min). Average intensity projections of the stacks were assembled into QuickTime movies (10 fps, 150x speed).

### Oris migration assay

The Oris™ 96-well cell migration assay kit (CMA1.101, Platypus Technologies) was used (3.5 × 10^4^ cells seeded/well). After plug removal, cells were treated without or with HGF (20 ng/ml) and PHA-665752 or ARQ197. Cell migration was monitored for 25 h using an automated ImageXpress Micro 2 (Molecular Devices) equipped with environmental control. Images were acquired at 5 h intervals with a 10× 0.2 NA Plan Apo objective (Nikon) and Roper CoolSNAP HQ camera (Roper Scientific). Wound closure was quantified using the threshold method in the MetaXpress software (Version MX 3.1.0.93).

### Matrigel invasion assay

A total of 25’000 cells were suspended in complete medium and seeded on the upper side of the Matrigel-coated membrane (BD 354480). Complete medium with or without 20 ng/ml HGF as used in the lower chamber. After 24 h, transmigrated cells were fixed with 4% PFA and stained with 0.05% crystal violet.

### Single cell motility assay

Cells were seeded on 96-well glass bottom plates (In Vitro Scientific)) at 40% confluency in assay medium with or without HGF (20 ng/mL) and cell motility was acquired using the ImageXpress Micro 2 microscope. Cell speed (total path length/time) was determined by manually tracking the cells at 5 min intervals for 6–18 h using ImageJ software (National Institutes of Health, USA).

### Flow cytometry

Cells were detached with Accutase (A6964, Sigma-Aldrich), fixed in 0.5% PFA for 10 min and washed in 0.5% Tween 20 (P9416, Sigma-Aldrich) and collected in flow cytometry (FC) buffer (5% FBS, 0.5% BSA, 0.1% Na-azide in PBS). 0.25 × 10^6^ cells per sample were stained with the following primary antibodies: CD44-Alexa488 (103016, 1:50), Isotype control-Alexa488 (400625, 1:50) (BioLegend), c-Met-biotin (13–8858, 1:100), c-Met (5631, 1:100) (Cell Signaling), Isotype control-biotin (13–4301, 1:100) and CD44v6 (BMS125, 1:100) (eBioscience), and Isotype control mouse IgG1 (02–6502, Life Technologies, 1:10 – 1:50). Secondary antibodies: anti-mouse-Alexa647 (A31571, Life Technologies, 1:10000) and Streptavidin-PE (12–4317, eBioscience, 1:10000). Sequential incubations (double staining) were interrupted by three washes. Sample acquisition (10000 events) in BDFACSCanto II flow cytometer (BD Bioscience).

### RNA expression analysis by qRT-PCR

Total RNA was extracted using the RNeasy Mini Kit (Qiagen, Basel, Switzerland) following the manufacturer’s instructions 1 μg of total RNA was used as template for reverse transcription, which was triggered by random hexamer primers and performed by using the High-Capacity cDNA Reverse Transcription Kit (Applied Biosystems). qRT-PCR was performed under conditions optimized for the ABI7900HT instrument, using Gene Expression Master Mix (Applied Biosystems). Probe-primer specific for the following genes (purchased from Applied Biosystems) were used: c-Met (Hs HS01565584_m1), HGF (Hs00300159_m1), CD44 (HS01075854_m1), CD44v6 (Hs01075854_m1). The relative gene expression was calculated for each gene of interest by using the ∆∆CT method, where cycle threshold (CT) values were normalized to the housekeeping gene 18S (Hs03003631_g1) (Applied Biosystems).

### Microbead invasion assay

Approximately 500 Cytodex Microcarrier beads (Sigma Aldrich C3275) per 1.25 × 10^4^ LA-EGFP-DAOY cells/ml were mixed in FACS tubes (BD Falcon **T7597-5 J**) and incubated at 37°C for 6 h, followed by incubation under rotation for 18 h. Non-adherent cells were removed. Cell-coated microbeads were resuspended in 2.5% bovine collagen I (5005-B, Advanced BioMatrix) in 96-well plate, after polymerization of collagen overlaid with fresh medium and treated with appropriate concentrations of c-Met inhibitors or HGF. After 24 h, cells were fixed with 4% PFA and stained with Hoechst. Images were acquired using the ImageXpress microscope. The distance between the microbead and the nuclei of the invaded cells was measured using ImageJ software. Velocity was calculated as the distance of displacement/time.

### Generation of inducible cell lines

Inducible shRNA DAOY cell lines were generated by lentiviral transduction. Virus was produced in HEK293T using 4.5 μg of inducible pLV-H1TetO-RFP-Bsd vectors encoding either MAP4K4 shRNA (Biosettia, sh_NM_001242559 1–4) or scramble shRNA (Biosettia) along with lentivirus packaging plasmids pRev (1 μg), pMDL (3 μg), and pVSV (1.5 μg). Lentivirus-containing supernatants were added to recipient cells in the presence of 10 μg/ml of Polybrene (AL-118, Sigma,-Aldrich). At 48 h post-transduction, the culture medium was removed and stable cells were selected with 5 μg/ml blasticidin (15205, Sigma-Aldrich). Doxycycline-containing (Sigma, 44577) medium was added for 48 h for shRNA induction and protein downregulation was verified by IB and qRT-PCR.

### RNA interference

The cells were transfected using either Silencer Select siRNA specific for MAP4K4 (ID: 18096) or Silencer select negative control #1 (ID: 4390843) (Ambion). Each siRNA was used at the final concentration of 5 nM in combination with Dharmafect 4 transfection reagent (Dharmacon), according to the manufacturer’s instructions. MAP4K4 (ID: 18096) or Silencer select negative control #1 (ID: 4390843) (Ambion) were used. After 24, 48, and 72 h cells were harvested for both mRNA and protein extraction, to assess gene expression by qRT-PCR and protein content by immunoblotting.

### Statistical analysis

Data are represented as the mean ± SD. Statistical analyses were performed using one-way analysis of variance (ANOVA) followed by Bonferroni’s Multiple Comparisons test (for details please see Additional file 9: Table ST1) if not otherwise stated. P-values <0.05 were considered significant. [* < 0.05, ** < 0.01, *** < 0.001].

## Additional files

Additional file 1: Figure S1. c-Met and HGF is specifically increased in the SHH subgroup of medulloblastoma. Comparison of subgroup-specific expression of **(A)** c-Met, **(B)** HGF, **(C)** CD44, and **(D)** mitogen-activated protein kinase kinase kinase kinase 4 (MAP4K4) in the MAGIC (n = 285) and Northcott (n = 103) datasets. Box plots show median, mean (+), and whiskers: 5–95 percentile. Additional file 2: Figure S2. PHA-665752 and ARQ197 block proliferation/viability of medulloblastoma cells at low molar concentrations. DAOY and UW228 cells in medium containing 0% or 10% FCS were treated with PHA-665752 or ARQ197 as indicated. Proliferation and viability of the cells were measured using the WST assay at 0 h and after 24, 48, and 72 h. Additional file 3: Figure S3. c-Met inhibitors block basal and HGF-induced gap closure in medium containing 10% fetal calf serum (FCS). (A) Oris migration assays using DAOY or UW228 cells in 10% FCS-containing medium treated with HGF (20 ng/mL) and c-Met inhibitors PHA-665752 and ARQ 197 (125 nM). Progression of gap closure over time expressed as area in pixels covered by cells is shown. **(B)** As A) but progression of gap closure shown for 0–10 h only. Additional file 4: Figure S4. Pharmacological JNK inhibition blocks HGF-induced motility. Speed of single cells in the absence or presence of HGF (20 ng/mL) and the JNK inhibitors AEG 3482 (5 μM) and JIP-1 (10 μM) was acquired using live cell microscopy imaging. Path lengths of individual cells after 18 h are shown (bars = means). Additional file 5: Figure S5.
**(A)** IFA of c-Met and p-c-Met localization in lamellipodia of UW228 cells. Color overlay and inverted grey-scale images of p-c-Met (red), F-actin (green), and c-Met (blue) are shown. Magnifications are 4× of boxed area. Arrows indicate c-Met-rich lamellipodia. **(B)** Still images of representative cells from movies. Panels to the right of each image show kymographic analysis of protrusion along lines perpendicular to the cortical F-actin. **C)** Immunoblotting analysis of stable, doxycycline-inducible DAOY shControl (scrambled) and shMAP4K4_3 and shMAP4K4_3 cell lines after 48 h doxycycline treatment using concentrations as indicated. Additional file 6:
**SM1F-actin dynamics in UW228 cells expressing Lifeact (LA)-enhanced green fluorescent protein (EGFP. 15 min recording time, 10 frames per second (fps), acceleration 150x.).**
Additional file 7:
**SM2F-actin dynamics in HGF-stimulated (20 ng/ml, 3 h) UW228 cells expressing LA-EGFP.** 15 min recording time, 10 frames per second (fps), acceleration 150x. Additional file 8:
**SM3F-actin dynamics in HGF-stimulated (20 ng/ml, 3 h) UW228 cells expressing LA-EGFP treated with PHA (250 nM).** 15 min recording time, 10 frames per second (fps), acceleration 150x. Additional file 9:
**ST1List of statistical analyses performed.**

## Abbreviations

2D/3D: Two- and three-dimensional
c-Met: Mesenchymal epithelial transition factor
ERK: Extracellular-signal-regulated kinase
JNK: c-Jun N-terminal kinase
HA: Hyaluronan
HGF: Hepatocyte growth factor/scatter factor
MAP4K4: Mitogen-activated protein kinase kinase kinase kinase 4
Myc: v-myc avian myelocytomatosis viral oncogene homolog
MYCN: Avian myelocytomatosis viral oncogene neuroblastoma derived homolog
SHH: Sonic hedgehog
WNT: Wingless
