# Supplementary figures and images for: The Ser/Thr kinase MAP4K4 drives c-Met-induced motility and invasiveness in a cell-based model of SHH medulloblastoma
Source: Springerplus. 2015 Jan 14;4:19. doi: 10.1186/s40064-015-0784-2 (PMC4302160; doi:10.1186/s40064-015-0784-2)

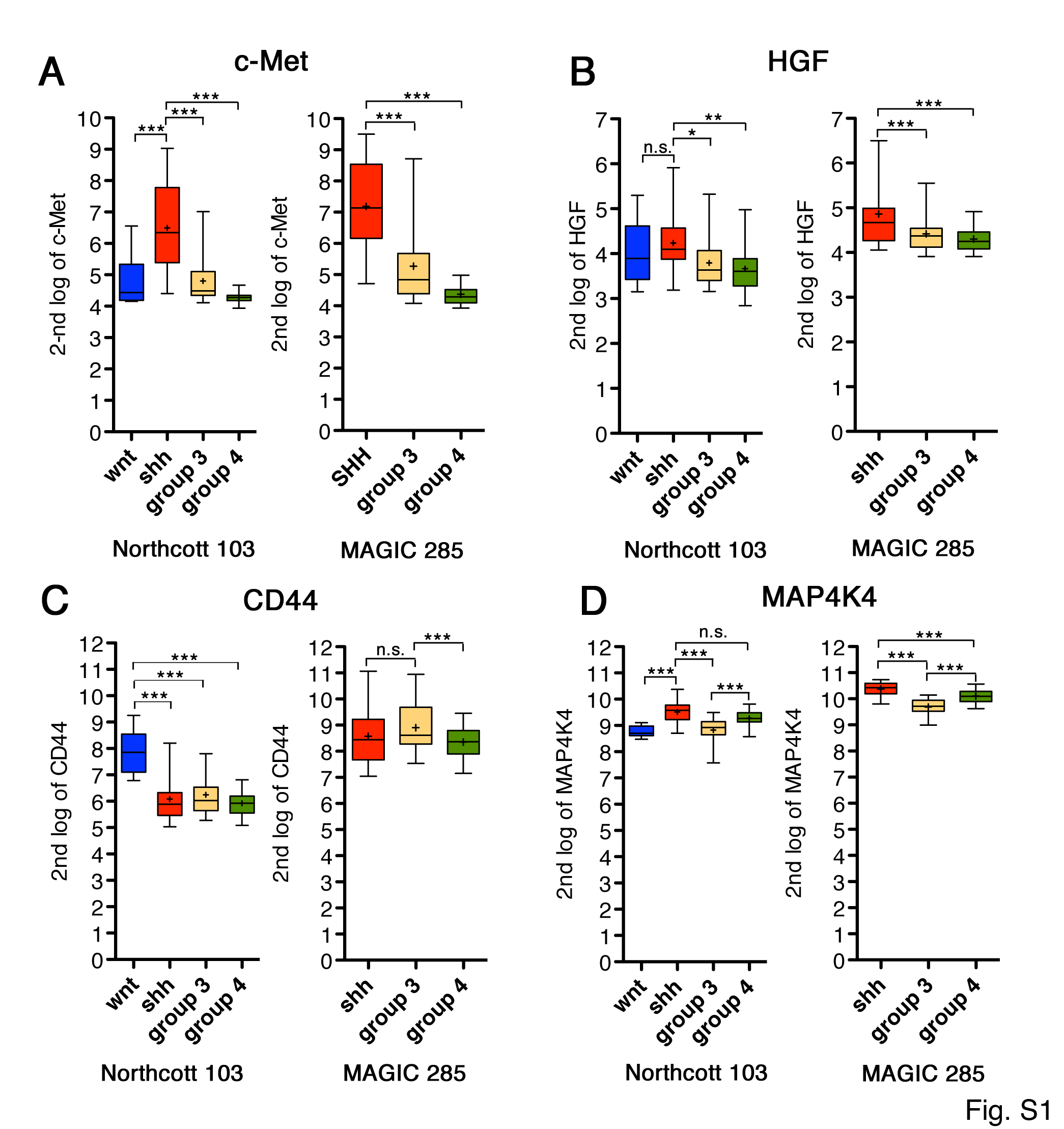

Supplement: Additional file 1: Figure S1. — c-Met and HGF is specifically increased in the SHH subgroup of medulloblastoma. Comparison of subgroup-specific expression of (A) c-Met, (B) HGF, (C) CD44, and (D) mitogen-activated protein kinase kinase kinase kinase 4 (MAP4K4) in the MAGIC (n = 285) and Northcott (n = 103) datasets. Box plots show median, mean (+), and whiskers: 5–95 percentile. [file 40064_2015_784_MOESM1_ESM.tiff]

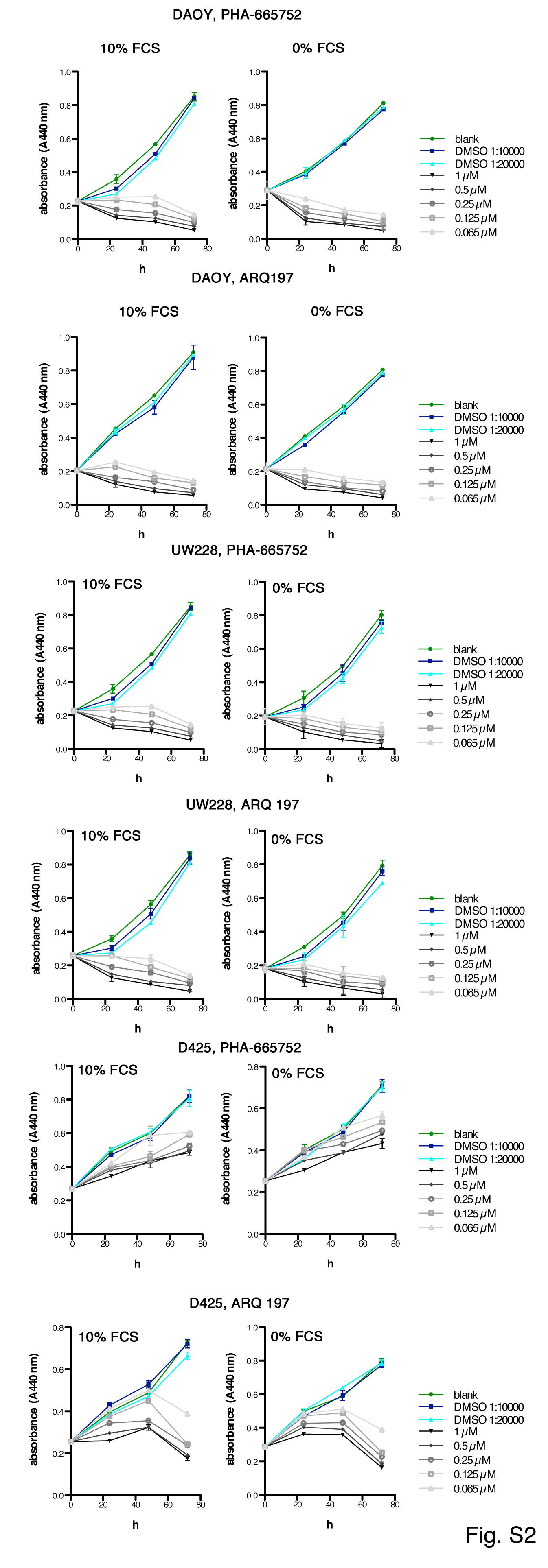

Supplement: Additional file 2: Figure S2. — PHA-665752 and ARQ197 block proliferation/viability of medulloblastoma cells at low molar concentrations. DAOY and UW228 cells in medium containing 0% or 10% FCS were treated with PHA-665752 or ARQ197 as indicated. Proliferation and viability of the cells were measured using the WST assay at 0 h and after 24, 48, and 72 h. [file 40064_2015_784_MOESM2_ESM.tiff]

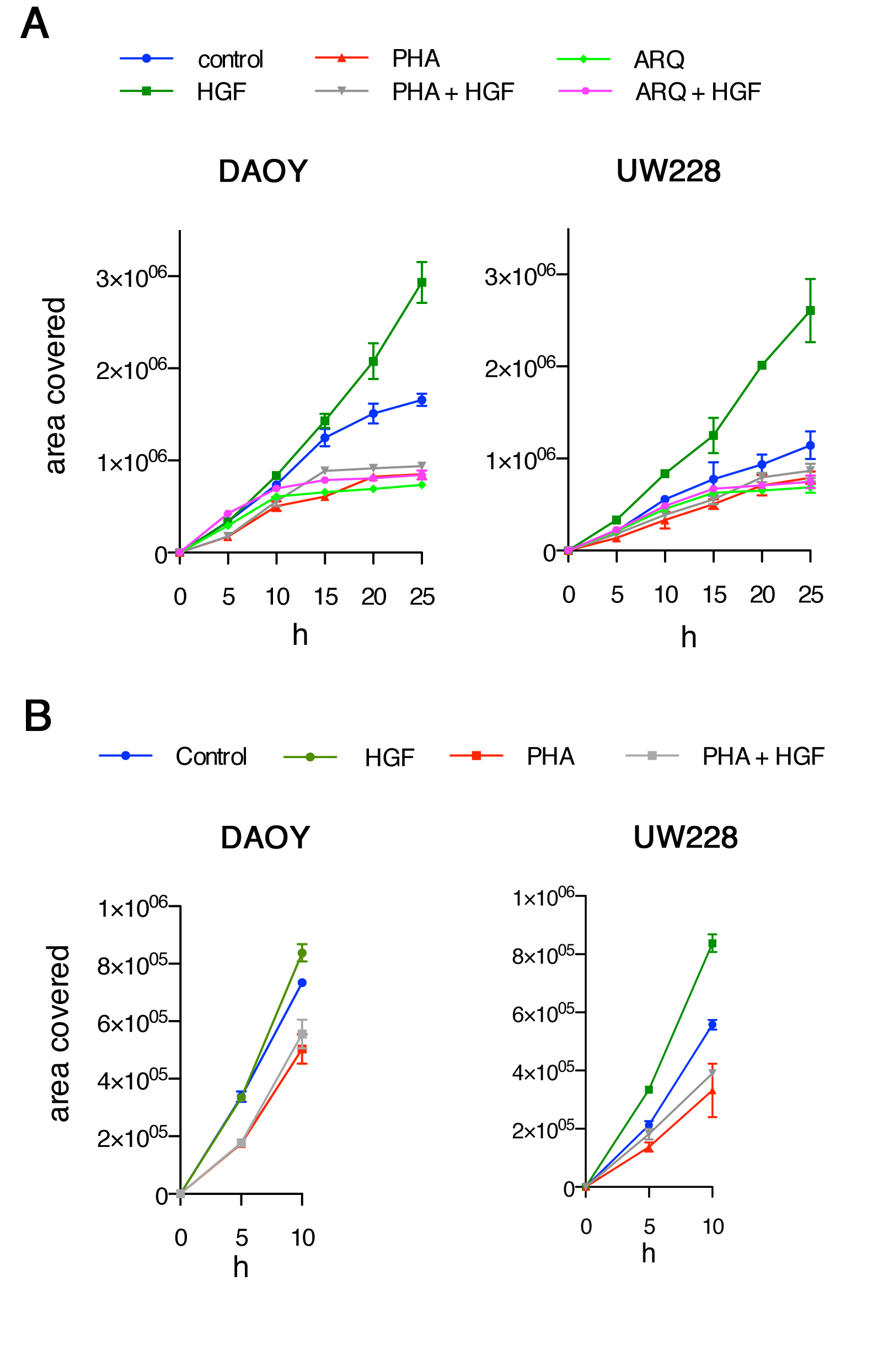

Supplement: Additional file 3: Figure S3. — c-Met inhibitors block basal and HGF-induced gap closure in medium containing 10% fetal calf serum (FCS). (A) Oris migration assays using DAOY or UW228 cells in 10% FCS-containing medium treated with HGF (20 ng/mL) and c-Met inhibitors PHA-665752 and ARQ 197 (125 nM). Progression of gap closure over time expressed as area in pixels covered by cells is shown. (B) As A) but progression of gap closure shown for 0–10 h only. [file 40064_2015_784_MOESM3_ESM.tiff]

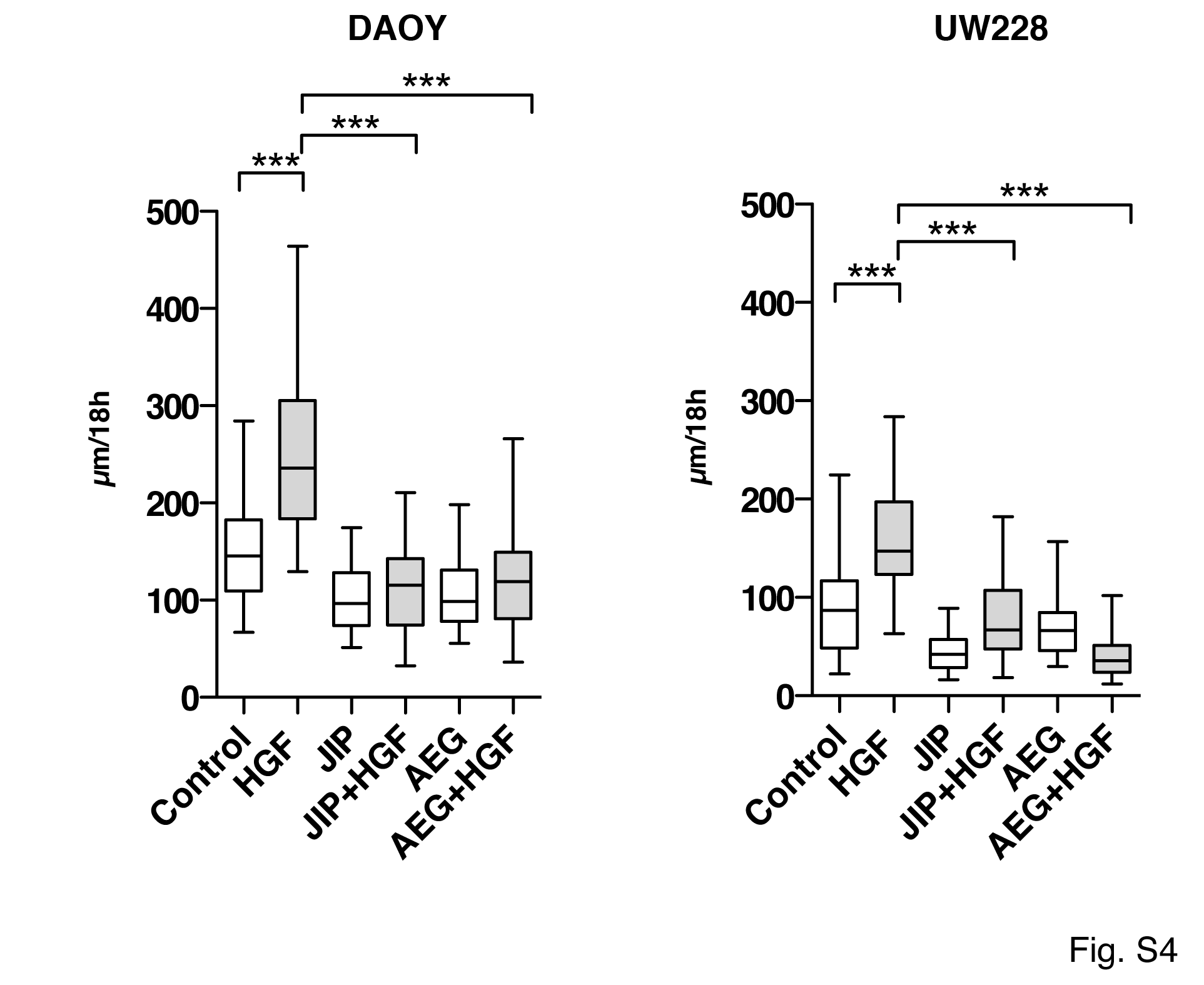

Supplement: Additional file 4: Figure S4. — Pharmacological JNK inhibition blocks HGF-induced motility. Speed of single cells in the absence or presence of HGF (20 ng/mL) and the JNK inhibitors AEG 3482 (5 μM) and JIP-1 (10 μM) was acquired using live cell microscopy imaging. Path lengths of individual cells after 18 h are shown (bars = means). [file 40064_2015_784_MOESM4_ESM.tiff]

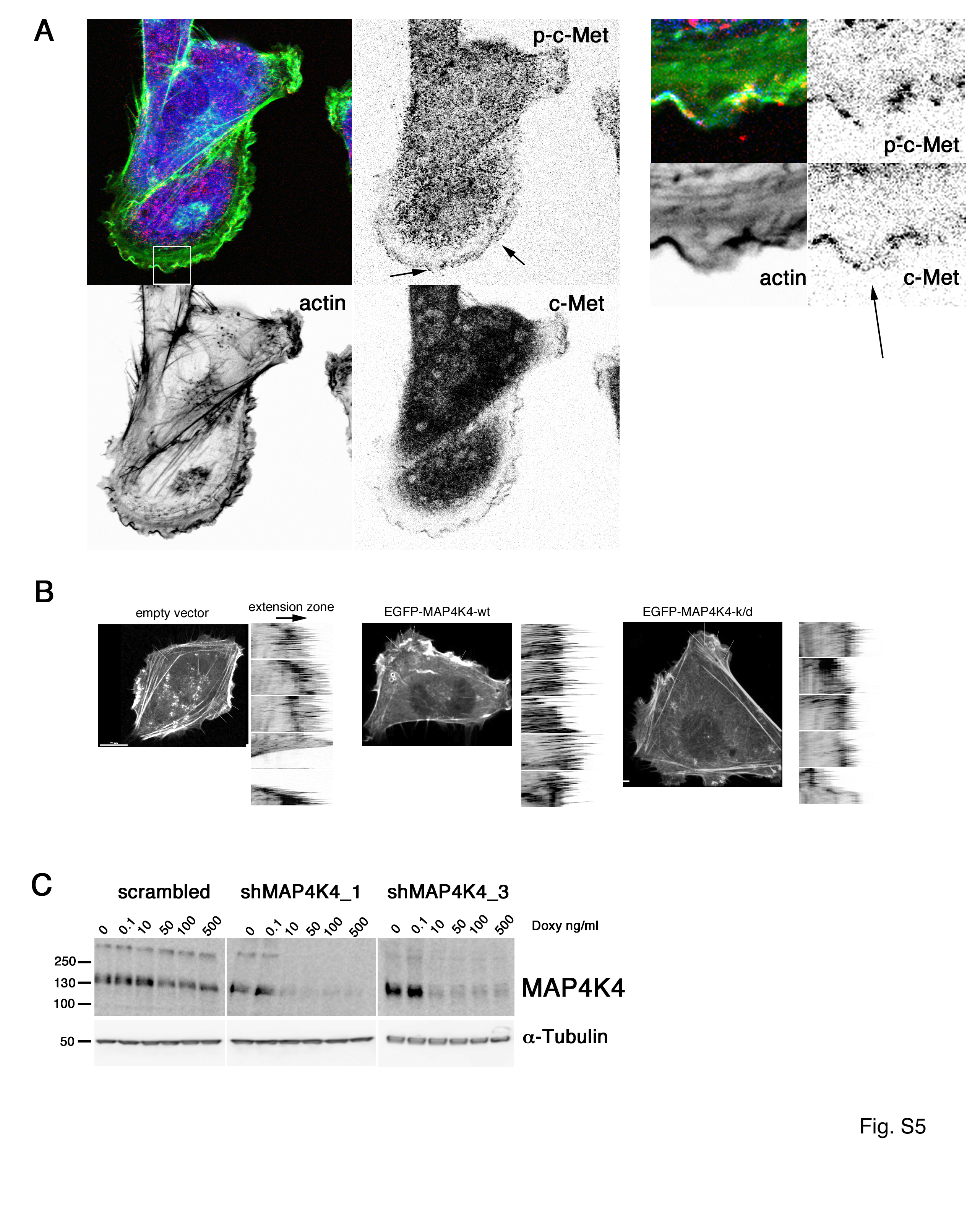

Supplement: Additional file 5: Figure S5. — (A) IFA of c-Met and p-c-Met localization in lamellipodia of UW228 cells. Color overlay and inverted grey-scale images of p-c-Met (red), F-actin (green), and c-Met (blue) are shown. Magnifications are 4× of boxed area. Arrows indicate c-Met-rich lamellipodia. (B) Still images of representative cells from movies. Panels to the right of each image show kymographic analysis of protrusion along lines perpendicular to the cortical F-actin. C) Immunoblotting analysis of stable, doxycycline-inducible DAOY shControl (scrambled) and shMAP4K4_3 and shMAP4K4_3 cell lines after 48 h doxycycline treatment using concentrations as indicated. [file 40064_2015_784_MOESM5_ESM.tiff]
