# Supplementary material for: The Ser/Thr kinase MAP4K4 drives c-Met-induced motility and invasiveness in a cell-based model of SHH medulloblastoma
Source: Springerplus. 2015 Jan 14;4:19. doi: 10.1186/s40064-015-0784-2 (PMC4302160; doi:10.1186/s40064-015-0784-2)
Supplement: Additional file 9: — ST1List of statistical analyses performed. [file 40064_2015_784_MOESM9_ESM.pdf]

**Fig. 1A, c-Met high**					
Table Analyzed	c-Met high than av. cereb.				
One-way analysis of variance					
P value	0.0007				
P value summary	***				
Are means signif. different? (P < 0.05)	Yes				
Number of groups	4				
F	6.977				
R square	0.3552				
Bartlett's test for equal variances					
Bartlett's statistic (corrected)	6.464				
P value	0.0911				
P value summary	ns				
Do the variances differ signif. (P < 0.05)	No				
ANOVA Table	SS	df	MS		
Treatment (between columns)	18.93	3	6.31		
Residual (within columns)	34.37	38	0.9044		
Total	53.29	41			
Bonferroni's Multiple Comparison Test	Mean Diff.	t	Significant? P < 0.05?	Summary	95% CI of diff
Nor. cereb. - Roth - 9 - vs MB Gilbertson	-1.664	3.968	Yes	**	-2.831 to -0.4967
Nor. cereb. - Roth - 9 - vs MB Kool	-1.726	4.117	Yes	**	-2.894 to -0.5592
Nor. cereb. - Roth - 9 - vs MB Delattre	-1.35	3.011	Yes	*	-2.598 to -0.1022
MB Gilbertson vs MB Kool	-0.0625	0.161	No	ns	-1.143 to 1.018
MB Gilbertson vs MB Delattre	0.3139	0.7485	No	ns	-0.8533 to 1.481
MB Kool vs MB Delattre	0.3764	0.8976	No	ns	-0.7908 to 1.544

**Fig. 1A, CD44**					
Table Analyzed	CD44				
One-way analysis of variance					
P value	0.0066				
P value summary	**				
Are means signif. different? (P < 0.05)	Yes				
Number of groups	4				
F	4.194				
R square	0.05919				
Bartlett's test for equal variances					
Bartlett's statistic (corrected)	7.896				
P value	0.0482				
P value summary	*				
Do the variances differ signif. (P < 0.05)	Yes				
ANOVA Table	SS	df	MS		
Treatment (between columns)	22.29	3	7.431		
Residual (within columns)	354.3	200	1.772		
Total	376.6	203			
Bonferroni's Multiple Comparison Test	Mean Diff.	t	Significant? P < 0.05?	Summary	95% CI of diff
Nor. cereb. - Roth - 9 - vs MB Gilbertson	-1.574	3.355	Yes	**	-2.825 to -0.3239
Nor. cereb. - Roth - 9 - vs MB Kool	-1.419	2.99	Yes	*	-2.685 to -0.1543
Nor. cereb. - Roth - 9 - vs MB Delattre	-1.651	3.459	Yes	**	-2.924 to -0.3792
MB Gilbertson vs MB Kool	0.1548	0.6795	No	ns	-0.4522 to 0.7618
MB Gilbertson vs MB Delattre	-0.07719	0.331	No	ns	-0.6987 to 0.5443
MB Kool vs MB Delattre	-0.232	0.9497	No	ns	-0.8828 to 0.4189

**Fig. 3A, DAOY**					
Table Analyzed	DAOY				
One-way analysis of variance					
P value	< 0.0001				
P value summary	***				
Are means signif. different? (P < 0.05)	Yes				
Number of groups	6				
F	48.11				
R square	0.5091				
Bartlett's test for equal variances					
Bartlett's statistic (corrected)	83.43				
P value	< 0.0001				
P value summary	***				
Do the variances differ signif. (P < 0.05)	Yes				
ANOVA Table	SS	df	MS		
Treatment (between columns)	1732000	5	346303		
Residual (within columns)	1670000	232	7198		
Total	3401000	237			
Bonferroni's Multiple Comparison Test	Mean Diff.	t	Significant? P < 0.05?	Summary	95% CI of diff
Control vs HGF 20 ng/ml	-196.7	10.24	Yes	***	-253.7 to -139.7
Control vs PHA	44.98	2.371	No	ns	-11.29 to 101.2
Control vs PHA+HGF	60.23	3.175	Yes	*	3.969 to 116.5
Control vs ARQ	0.562	0.02962	No	ns	-55.70 to 56.83
Control vs ARQ+HGF	34.99	1.844	No	ns	-21.28 to 91.25
HGF 20 ng/ml vs PHA	241.7	12.58	Yes	***	184.7 to 298.7
HGF 20 ng/ml vs PHA+HGF	257	13.37	Yes	***	200.0 to 314.0
HGF 20 ng/ml vs ARQ	197.3	10.27	Yes	***	140.3 to 254.3
HGF 20 ng/ml vs ARQ+HGF	231.7	12.06	Yes	***	174.7 to 288.7
PHA vs PHA+HGF	15.26	0.8041	No	ns	-41.01 to 71.52
PHA vs ARQ	-44.42	2.341	No	ns	-100.7 to 11.85
PHA vs ARQ+HGF	-9.993	0.5268	No	ns	-66.26 to 46.27
PHA+HGF vs ARQ	-59.67	3.145	Yes	*	-115.9 to -3.407
PHA+HGF vs ARQ+HGF	-25.25	1.331	No	ns	-81.51 to 31.02
ARQ vs ARQ+HGF	34.42	1.815	No	ns	-21.84 to 90.69

Fig. 3A, UW228					
Table Analyzed	UW228				
One-way analysis of variance					
P value	< 0.0001				
P value summary	***				
Are means signif. different? (P < 0.05)	Yes				
Number of groups	6				
F	29.99				
R square	0.4503				
Bartlett's test for equal variances					
Bartlett's statistic (corrected)	61.16				
P value	< 0.0001				
P value summary	***				
Do the variances differ signif. (P < 0.05)	Yes				
ANOVA Table	SS	df	MS		
Treatment (between columns)	458611	5	91722		
Residual (within columns)	559740	183	3059		
Total	1018000	188			
Bonferroni's Multiple Comparison Test	Mean Diff.	t	Significant? P < 0.05?	Summary	95% CI of diff
Control vs HGF 20 ng/ml	-78.43	5.583	Yes	***	-120.2 to -36.65
Control vs PHA	59.21	4.248	Yes	***	17.76 to 100.7
Control vs PHA+HGF	54.53	3.912	Yes	**	13.08 to 95.98
Control vs ARQ	50.59	3.63	Yes	**	9.133 to 92.04
Control vs ARQ+HGF	51.5	3.666	Yes	**	9.723 to 93.28
HGF 20 ng/ml vs PHA	137.6	9.876	Yes	***	96.19 to 179.1
HGF 20 ng/ml vs PHA+HGF	133	9.54	Yes	***	91.51 to 174.4
HGF 20 ng/ml vs ARQ	129	9.257	Yes	***	87.56 to 170.5
HGF 20 ng/ml vs ARQ+HGF	129.9	9.25	Yes	***	88.15 to 171.7
PHA vs PHA+HGF	-4.679	0.3384	No	ns	-45.80 to 36.44
PHA vs ARQ	-8.623	0.6237	No	ns	-49.75 to 32.50
PHA vs ARQ+HGF	-7.706	0.5529	No	ns	-49.16 to 33.75
PHA+HGF vs ARQ	-3.944	0.2852	No	ns	-45.07 to 37.18
PHA+HGF vs ARQ+HGF	-3.026	0.2171	No	ns	-44.48 to 38.43
ARQ vs ARQ+HGF	0.9173	0.06582	No	ns	-40.54 to 42.37

Fig. 3B DAOY			
Table Analyzed	DAOY		
Column A	Control		
vs	vs		
Column B	HGF 20ng/ml		
Unpaired t test			
P value	0.0038		
P value summary	**		
Are means signif. different? (P < 0.05)	Yes		
One- or two-tailed P value?	Two-tailed		
t, df	t=6.025 df=4		
How big is the difference?			
Mean ± SEM of column A	122.0 ± 31.90 N=3		
Mean ± SEM of column B	370.7 ± 26.19 N=3		
Difference between means	-248.7 ± 41.27		
95% confidence interval	-363.2 to -134.1		
R square	0.9007		
F test to compare variances			
F,DFn, Dfd	1.483, 2, 2		
P value	0.8056		
P value summary	ns		
Are variances significantly different?	No		
Fig. 3B UW228			
Table Analyzed	UW228		
Column A	Control		
vs	vs		
Column B	HGF		
Unpaired t test			
P value	0.0454		
P value summary	*		
Are means signif. different? (P < 0.05)	Yes		
One- or two-tailed P value?	Two-tailed		
t, df	t=2.872 df=4		
How big is the difference?			
Mean ± SEM of column A	6.000 ± 2.517 N=3		
Mean ± SEM of column B	60.33 ± 18.75 N=3		
Difference between means	-54.33 ± 18.92		
95% confidence interval	-106.8 to -1.825		
R square	0.6735		
F test to compare variances			
F,DFn, Dfd	55.49, 2, 2		
P value	0.0354		
P value summary	*		
Are variances significantly different?	Yes		

Fig. 3D DAOY, Whiskers 5-95% percentile					
Table Analyzed	DAOY				
One-way analysis of variance					
P value	< 0.0001				
P value summary	***				
Are means signif. different? (P < 0.05)	Yes				
Number of groups	6				
F	48.11				
R square	0.5091				
Bartlett's test for equal variances					
Bartlett's statistic (corrected)	83.43				
P value	< 0.0001				
P value summary	***				
Do the variances differ signif. (P < 0.05)	Yes				
ANOVA Table	SS	df	MS		
Treatment (between columns)	1732000	5	346303		
Residual (within columns)	1670000	232	7198		
Total	3401000	237			
Bonferroni's Multiple Comparison Test	Mean Diff.	t	Significant? P < 0.05?	Summary	95% CI of diff
Control vs HGF 20 ng/ml	-196.7	10.24	Yes	***	-253.7 to -139.7
Control vs PHA	44.98	2.371	No	ns	-11.29 to 101.2
Control vs PHA+HGF	60.23	3.175	Yes	*	3.969 to 116.5
Control vs ARQ	0.562	0.02962	No	ns	-55.70 to 56.83
Control vs ARQ+HGF	34.99	1.844	No	ns	-21.28 to 91.25
HGF 20 ng/ml vs PHA	241.7	12.58	Yes	***	184.7 to 298.7
HGF 20 ng/ml vs PHA+HGF	257	13.37	Yes	***	200.0 to 314.0
HGF 20 ng/ml vs ARQ	197.3	10.27	Yes	***	140.3 to 254.3
HGF 20 ng/ml vs ARQ+HGF	231.7	12.06	Yes	***	174.7 to 288.7
PHA vs PHA+HGF	15.26	0.8041	No	ns	-41.01 to 71.52
PHA vs ARQ	-44.42	2.341	No	ns	-100.7 to 11.85
PHA vs ARQ+HGF	-9.993	0.5268	No	ns	-66.26 to 46.27
PHA+HGF vs ARQ	-59.67	3.145	Yes	*	-115.9 to -3.407
PHA+HGF vs ARQ+HGF	-25.25	1.331	No	ns	-81.51 to 31.02
ARQ vs ARQ+HGF	34.42	1.815	No	ns	-21.84 to 90.69

Fig. 3D UW228, Whiskers 5-95% percentile					
Table Analyzed	UW228				
One-way analysis of variance					
P value	< 0.0001				
P value summary	***				
Are means signif. different? (P < 0.05)	Yes				
Number of groups	6				
F	29.99				
R square	0.4503				
Bartlett's test for equal variances					
Bartlett's statistic (corrected)	61.16				
P value	< 0.0001				
P value summary	***				
Do the variances differ signif. (P < 0.05)	Yes				
ANOVA Table	SS	df	MS		
Treatment (between columns)	458611	5	91722		
Residual (within columns)	559740	183	3059		
Total	1018000	188			
Bonferroni's Multiple Comparison Test	Mean Diff.	t	Significant? P < 0.05?	Summary	95% CI of diff
Control vs HGF 20 ng/ml	-78.43	5.583	Yes	***	-120.2 to -36.65
Control vs PHA	59.21	4.248	Yes	***	17.76 to 100.7
Control vs PHA+HGF	54.53	3.912	Yes	**	13.08 to 95.98
Control vs ARQ	50.59	3.63	Yes	**	9.133 to 92.04
Control vs ARQ+HGF	51.5	3.666	Yes	**	9.723 to 93.28
HGF 20 ng/ml vs PHA	137.6	9.876	Yes	***	96.19 to 179.1
HGF 20 ng/ml vs PHA+HGF	133	9.54	Yes	***	91.51 to 174.4
HGF 20 ng/ml vs ARQ	129	9.257	Yes	***	87.56 to 170.5
HGF 20 ng/ml vs ARQ+HGF	129.9	9.25	Yes	***	88.15 to 171.7
PHA vs PHA+HGF	-4.679	0.3384	No	ns	-45.80 to 36.44
PHA vs ARQ	-8.623	0.6237	No	ns	-49.75 to 32.50
PHA vs ARQ+HGF	-7.706	0.5529	No	ns	-49.16 to 33.75
PHA+HGF vs ARQ	-3.944	0.2852	No	ns	-45.07 to 37.18
PHA+HGF vs ARQ+HGF	-3.026	0.2171	No	ns	-44.48 to 38.43
ARQ vs ARQ+HGF	0.9173	0.06582	No	ns	-40.54 to 42.37

Fig. 4A, DAOY, Whiskers 5-95% percentile					
Table Analyzed	DAOY				
One-way analysis of variance					
P value	< 0.0001				
P value summary	***				
Are means signif. different? (P < 0.05)	Yes				
Number of groups	4				
F	33.35				
R square	0.3907				
Bartlett's test for equal variances					
Bartlett's statistic (corrected)	22.28				
P value	< 0.0001				
P value summary	***				
Do the variances differ signif. (P < 0.05)	Yes				
ANOVA Table	SS	df	MS		
Treatment (between columns)	371761	3	123920		

Residual (within columns)	579720	156	3716		
Total	951481	159			
Bonferroni's Multiple Comparison Test	Mean Diff.	t	Significant? P < 0.05?	Summary	95% CI of diff
Control vs SP600125	6.375	0.4677	No	ns	-30.05 to 42.80
Control vs HGF 20 ng/ml	-114.1	8.368	Yes	***	-150.5 to -77.64
Control vs HGF+SP600125	-23.92	1.754	No	ns	-60.34 to 12.51
SP600125 vs HGF 20 ng/ml	-120.4	8.836	Yes	***	-156.9 to -84.02
SP600125 vs HGF+SP600125	-30.29	2.222	No	ns	-66.72 to 6.137
HGF 20 ng/ml vs HGF+SP600125	90.15	6.614	Yes	***	53.73 to 126.6

**Fig. 4A, UW228 Whiskers 5-95% percentile**					
Table Analyzed	UW228				
One-way analysis of variance					
P value	< 0.0001				
P value summary	***				
Are means signif. different? (P < 0.05)	Yes				
Number of groups	4				
F	53.7				
R square	0.5793				
Bartlett's test for equal variances					
Bartlett's statistic (corrected)	51.69				
P value	< 0.0001				
P value summary	***				
Do the variances differ signif. (P < 0.05)	Yes				
ANOVA Table	SS	df	MS		
Treatment (between columns)	385444	3	128481		
Residual (within columns)	279906	117	2392		
Total	665351	120			
Bonferroni's Multiple Comparison Test	Mean Diff.	t	Significant? P < 0.05?	Summary	95% CI of diff
Control vs SP600125	40.79	3.256	Yes	**	7.166 to 74.41
Control vs HGF 20 ng/ml	-103.4	8.251	Yes	***	-137.0 to -69.74
Control vs HGF+SP600125	28.19	2.25	No	ns	-5.434 to 61.81
SP600125 vs HGF 20 ng/ml	-144.1	11.41	Yes	***	-178.0 to -110.3
SP600125 vs HGF+SP600125	-12.6	0.9977	No	ns	-46.49 to 21.30
HGF 20 ng/ml vs HGF+SP600125	131.5	10.42	Yes	***	97.65 to 165.4

**Fig. 5C**					
Table Analyzed	Data 1				
One-way analysis of variance					
P value	< 0.0001				
P value summary	***				
Are means signif. different? (P < 0.05)	Yes				
Number of groups	3				
F	41.95				
R square	0.3203				
Bartlett's test for equal variances					
Bartlett's statistic (corrected)	86.25				
P value	< 0.0001				
P value summary	***				
Do the variances differ signif. (P < 0.05)	Yes				
ANOVA Table	SS	df	MS		
Treatment (between columns)	19698	2	9849		
Residual (within columns)	41795	178	234.8		
Total	61493	180			
Bonferroni's Multiple Comparison Test	Mean Diff.	t	Significant? P < 0.05?	Summary	95% CI of diff
control vs MAP4K4-wt	-18.87	6.511	Yes	***	-25.87 to -11.86
control vs MAP4K4-K/R	6.011	2.202	No	ns	-0.5865 to 12.61
MAP4K4-wt vs MAP4K4-K/R	24.88	8.935	Yes	***	18.15 to 31.61

**Fig. 5E**					
Table Analyzed	Data 1				
One-way analysis of variance					
P value	< 0.0001				
P value summary	***				
Are means signif. different? (P < 0.05)	Yes				
Number of groups	4				
F	52.67				
R square	0.334				
Bartlett's test for equal variances					
Bartlett's statistic (corrected)	28.22				
P value	< 0.0001				
P value summary	***				
Do the variances differ signif. (P < 0.05)	Yes				
ANOVA Table	SS	df	MS		
Treatment (between columns)	496400000	3	165500000		
Residual (within columns)	989600000	315	3142000		
Total	1486000000	318			
Bonferroni's Multiple Comparison Test	Mean Diff.	t	Significant? P < 0.05?	Summary	95% CI of diff
begin, scr vs end, scr	2368	8.369	Yes	***	1616 to 3119
begin, scr vs begin, shMAP4K4	-746.6	2.782	Yes	*	-1459 to -34.12
begin, scr vs end shMAP4K4	-536	1.785	No	ns	-1333 to 261.2
end, scr vs begin, shMAP4K4	-3114	11.65	Yes	***	-3824 to -2404
end, scr vs end shMAP4K4	-2904	9.698	Yes	***	-3698 to -2109
begin, shMAP4K4 vs end shMAP4K4	210.6	0.7373	No	ns	-547.8 to 969.0

**Fig. 5E**	
Table Analyzed	Data 2
Column A	shScr
vs	vs
Column B	shMAP4K4
Mann Whitney test	
P value	0.0208
Exact or approximate P value?	Gaussian Approximation
P value summary	*
Are medians signif. different? (P < 0.05)	Yes
One- or two-tailed P value?	Two-tailed
Sum of ranks in column A,B	1573 , 1508
Mann-Whitney U	518

**Fig. S1A**					
Table Analyzed	Northcott 103, c-Met				
One-way analysis of variance					
P value	< 0.0001				
P value summary	***				
Are means signif. different? (P < 0.05)	Yes				
Number of groups	4				
F	34.19				
R square	0.5088				
Bartlett's test for equal variances					
Bartlett's statistic (corrected)	93.99				
P value	< 0.0001				
P value summary	***				
Do the variances differ signif. (P < 0.05)	Yes				
ANOVA Table	SS	df	MS		
Treatment (between columns)	85.45	3	28.48		
Residual (within columns)	82.49	99	0.8332		
Total	167.9	102			
Bonferroni's Multiple Comparison Test	Mean Diff.	t	Significant? P < 0.05?	Summary	95% CI of diff
wnt vs shh	-1.693	5.078	Yes	***	-2.590 to -0.7953
wnt vs group 3	-0.002143	0.006373	No	ns	-0.9075 to 0.9032
wnt vs group 4	0.5264	1.608	No	ns	-0.3548 to 1.408
shh vs group 3	1.691	7.048	Yes	***	1.045 to 2.336
shh vs group 4	2.219	9.771	Yes	***	1.608 to 2.831
group 3 vs group 4	0.5286	2.284	No	ns	-0.09452 to 1.152
Table Analyzed	MAGIC_c-Met				
One-way analysis of variance					
P value	< 0.0001				
P value summary	***				
Are means signif. different? (P < 0.05)	Yes				
Number of groups	3				
F	198.2				
R square	0.5843				
Bartlett's test for equal variances					
Bartlett's statistic (corrected)	126.2				
P value	< 0.0001				
P value summary	***				
Do the variances differ signif. (P < 0.05)	Yes				
ANOVA Table	SS	df	MS		
Treatment (between columns)	321.2	2	160.6		
Residual (within columns)	228.5	282	0.8102		
Total	549.6	284			
Bonferroni's Multiple Comparison Test	Mean Diff.	t	Significant? P < 0.05?	Summary	95% CI of diff
SHH vs group 3	1.914	10.46	Yes	***	1.473 to 2.355
SHH vs group 4	2.814	19.8	Yes	***	2.472 to 3.156
group 3 vs group 4	0.8998	6.077	Yes	***	0.5433 to 1.256

**Fig. S1B**					
Table Analyzed	Northcott 103, HGF				
One-way analysis of variance					
P value	0.002				
P value summary	**				
Are means signif. different? (P < 0.05)	Yes				
Number of groups	4				
F	5.315				
R square	0.1399				
R square	0.1399				
Bartlett's test for equal variances					
Bartlett's statistic (corrected)	4.368				
P value	0.2244				
P value summary	ns				
Do the variances differ signif. (P < 0.05)	No				
ANOVA Table	SS	df	MS		
Treatment (between columns)	6.115	3	2.038		
Residual (within columns)	37.58	98	0.3835		
Total	43.7	101			

Bonferroni's Multiple Comparison Test	Mean Diff.	t	Significant? P < 0.05?	Summary	95% CI of diff
wnt vs shh	-0.2131	0.8732	No	ns	-0.8703 to 0.4440
wnt vs group 3	0.231	0.9225	No	ns	-0.4432 to 0.9052
wnt vs group 4	0.3604	1.485	No	ns	-0.2931 to 1.014
shh vs group 3	0.4441	2.735	Yes	*	0.006787 to 0.8813
shh vs group 4	0.5735	3.817	Yes	**	0.1688 to 0.9781
group 3 vs group 4	0.1294	0.8071	No	ns	-0.3023 to 0.5611

Table Analyzed	MAGIC_HGF				
One-way analysis of variance					
P value	< 0.0001				
P value summary	***				
Are means signif. different? (P < 0.05)	Yes				
Number of groups	3				
F	27.1				
R square	0.1612				
Bartlett's test for equal variances					
Bartlett's statistic (corrected)	97.92				
P value	< 0.0001				
P value summary	***				
Do the variances differ signif. (P < 0.05)	Yes				
ANOVA Table	SS	df	MS		
Treatment (between columns)	12.47	2	6.236		
Residual (within columns)	64.89	282	0.2301		
Total	77.37	284			
Bonferroni's Multiple Comparison Test	Mean Diff.	t	Significant? P < 0.05?	Summary	95% CI of diff
shh vs group 3	0.4445	4.557	Yes	***	0.2096 to 0.6794
shh vs group 4	0.5575	7.361	Yes	***	0.3751 to 0.7399
group 3 vs group 4	0.1131	1.433	No	ns	-0.07698 to 0.3031

**Fig. S1C**					
Table Analyzed	Northcott 103, CD44				
One-way analysis of variance					
P value	< 0.0001				
P value summary	***				
Are means signif. different? (P < 0.05)	Yes				
Number of groups	4				
F	15.32				
R square	0.3215				
Bartlett's test for equal variances					
Bartlett's statistic (corrected)	10.91				
P value	0.0122				
P value summary	*				
Do the variances differ signif. (P < 0.05)	Yes				
ANOVA Table	SS	df	MS		
Treatment (between columns)	24.38	3	8.126		
Residual (within columns)	51.45	97	0.5305		
Total	75.83	100			
Bonferroni's Multiple Comparison Test	Mean Diff.	t	Significant? P < 0.05?	Summary	95% CI of diff
wnt vs shh	1.748	6.091	Yes	***	0.9752 to 2.521
wnt vs group 3	1.588	5.392	Yes	***	0.7946 to 2.381
wnt vs group 4	1.908	6.665	Yes	***	1.137 to 2.679
shh vs group 3	-0.1606	0.8411	No	ns	-0.6751 to 0.3538
shh vs group 4	0.1593	0.8951	No	ns	-0.3201 to 0.6387
group 3 vs group 4	0.32	1.686	No	ns	-0.1911 to 0.8310

Table Analyzed	MAGIC_CD44				
One-way analysis of variance					
P value	0.0004				
P value summary	***				
Are means signif. different? (P < 0.05)	Yes				
Number of groups	3				
F	8.031				
R square	0.05389				
Bartlett's test for equal variances					
Bartlett's statistic (corrected)	25.77				
P value	< 0.0001				
P value summary	***				
Do the variances differ signif. (P < 0.05)	Yes				
ANOVA Table	SS	df	MS		
Treatment (between columns)	11.53	2	5.763		
Residual (within columns)	202.4	282	0.7176		
Total	213.9	284			
Bonferroni's Multiple Comparison Test	Mean Diff.	t	Significant? P < 0.05?	Summary	95% CI of diff
shh vs group 3	-0.3306	1.919	No	ns	-0.7455 to 0.08422
shh vs group 4	0.2158	1.613	No	ns	-0.1063 to 0.5379
group 3 vs group 4	0.5464	3.921	Yes	***	0.2108 to 0.8820

**Fig. S1D**					
Table Analyzed	Northcott 103, MAP4K4				
One-way analysis of variance					
P value	< 0.0001				
P value summary	***				
Are means signif. different? (P < 0.05)	Yes				
Number of groups	4				
F	17.34				
R square	0.3444				
Bartlett's test for equal variances					
Bartlett's statistic (corrected)	8.585				
P value	0.0353				
P value summary	*				
Do the variances differ signif. (P < 0.05)	Yes				
ANOVA Table	SS	df	MS		
Treatment (between columns)	8.529	3	2.843		
Residual (within columns)	16.23	99	0.164		
Total	24.76	102			
Bonferroni's Multiple Comparison Test	Mean Diff.	t	Significant? P < 0.05?	Summary	95% CI of diff
wnt vs shh	-0.7403	4.639	Yes	***	-1.170 to -0.3107
wnt vs group 3	-0.06	0.3681	No	ns	-0.4988 to 0.3788
wnt vs group 4	-0.5017	3.162	Yes	*	-0.9289 to -0.07451
shh vs group 3	0.6803	6.474	Yes	***	0.3974 to 0.9632
shh vs group 4	0.2386	2.428	No	ns	-0.02592 to 0.5031
group 3 vs group 4	-0.4417	4.259	Yes	***	-0.7209 to -0.1625

Table Analyzed	MAGIC-MAP4K4				
One-way analysis of variance					
P value	< 0.0001				
P value summary	***				
Are means signif. different? (P < 0.05)	Yes				
Number of groups	3				
F	66.22				
R square	0.3203				
Bartlett's test for equal variances					
Bartlett's statistic (corrected)	1.147				
P value	0.5635				
P value summary	ns				
Do the variances differ signif. (P < 0.05)	No				
ANOVA Table	SS	df	MS		
Treatment (between columns)	11.44	2	5.722		
Residual (within columns)	24.28	281	0.08641		
Total	35.73	283			
Bonferroni's Multiple Comparison Test	Mean Diff.	t	Significant? P < 0.05?	Summary	95% CI of diff
shh vs group 3	0.6842	11.39	Yes	***	0.5396 to 0.8288
shh vs group 4	0.2678	5.726	Yes	***	0.1552 to 0.3805
group 3 vs group 4	-0.4164	8.611	Yes	***	-0.5328 to -0.2999

**Fig. S4**					
Table Analyzed	DAOY				
One-way analysis of variance					
P value	< 0.0001				
P value summary	***				
Are means signif. different? (P < 0.05)	Yes				
Number of groups	6				
F	32.76				
R square	0.4849				
Bartlett's test for equal variances					
Bartlett's statistic (corrected)	35.52				
P value	< 0.0001				
P value summary	***				
Do the variances differ signif. (P < 0.05)	Yes				
ANOVA Table	SS	df	MS		
Treatment (between columns)	491965	5	98393		
Residual (within columns)	522633	174	3004		
Total	1015000	179			
Bonferroni's Multiple Comparison Test	Mean Diff.	t	Significant? P < 0.05?	Summary	95% CI of diff
Control vs HGF	-101.8	7.192	Yes	***	-143.9 to -59.66
Control vs JIP	48.27	3.411	Yes	*	6.153 to 90.39
Control vs JIP+HGF	39.09	2.762	No	ns	-3.030 to 81.20
Control vs AEG	43.56	3.079	Yes	*	1.448 to 85.68
Control vs AEG+HGF	29.19	2.063	No	ns	-12.93 to 71.31
HGF vs JIP	150	10.6	Yes	***	107.9 to 192.2
HGF vs JIP+HGF	140.9	9.954	Yes	***	98.75 to 183.0
HGF vs AEG	145.3	10.27	Yes	***	103.2 to 187.5
HGF vs AEG+HGF	131	9.255	Yes	***	88.85 to 173.1
JIP vs JIP+HGF	-9.183	0.6489	No	ns	-51.30 to 32.93
JIP vs AEG	-4.705	0.3325	No	ns	-46.82 to 37.41
JIP vs AEG+HGF	-19.08	1.348	No	ns	-61.20 to 23.04
JIP+HGF vs AEG	4.478	0.3165	No	ns	-37.64 to 46.59
JIP+HGF vs AEG+HGF	-9.898	0.6995	No	ns	-52.01 to 32.22
AEG vs AEG+HGF	-14.38	1.016	No	ns	-56.49 to 27.74

Table Analyzed	UW228				
One-way analysis of variance					
P value	< 0.0001				
P value summary	***				
Are means signif. different? (P < 0.05)	Yes				
Number of groups	6				
F	41.99				
R square	0.5468				
Bartlett's test for equal variances					
Bartlett's statistic (corrected)	43.17				
P value	< 0.0001				
P value summary	***				
Do the variances differ signif. (P < 0.05)	Yes				
ANOVA Table	SS	df	MS		
Treatment (between columns)	281409	5	56282		
Residual (within columns)	233242	174	1340		
Total	514651	179			
Bonferroni's Multiple Comparison Test	Mean Diff.	t	Significant? P < 0.05?	Summary	95% CI of diff
Control vs HGF	-70.97	7.507	Yes	***	-99.10 to -42.83
Control vs JIP	45.2	4.781	Yes	***	17.06 to 73.34
Control vs JIP+HGF	10.3	1.09	No	ns	-17.83 to 38.44
Control vs AEG	19.96	2.111	No	ns	-8.179 to 48.09
Control vs AEG+HGF	47.38	5.012	Yes	***	19.25 to 75.52
HGF vs JIP	116.2	12.29	Yes	***	88.03 to 144.3
HGF vs JIP+HGF	81.27	8.597	Yes	***	53.13 to 109.4
HGF vs AEG	90.92	9.618	Yes	***	62.79 to 119.1
HGF vs AEG+HGF	118.4	12.52	Yes	***	90.21 to 146.5
JIP vs JIP+HGF	-34.9	3.691	Yes	**	-63.03 to -6.761
JIP vs AEG	-25.24	2.67	No	ns	-53.38 to 2.893
JIP vs AEG+HGF	2.184	0.2311	No	ns	-25.95 to 30.32
JIP+HGF vs AEG	9.654	1.021	No	ns	-18.48 to 37.79
JIP+HGF vs AEG+HGF	37.08	3.923	Yes	**	8.945 to 65.22
AEG vs AEG+HGF	27.43	2.901	No	ns	-0.7088 to 55.56
